# Molecular Genealogy of a Mongol Queen’s Family and Her Possible Kinship with Genghis Khan

**DOI:** 10.1371/journal.pone.0161622

**Published:** 2016-09-14

**Authors:** Gavaachimed Lkhagvasuren, Heejin Shin, Si Eun Lee, Dashtseveg Tumen, Jae-Hyun Kim, Kyung-Yong Kim, Kijeong Kim, Ae Ja Park, Ho Woon Lee, Mi Jin Kim, Jaesung Choi, Jee-Hye Choi, Na Young Min, Kwang-Ho Lee

**Affiliations:** 1 Department of Science of Cultural Heritage, Graduate School, Chung-Ang University, Seoul, Korea; 2 Department of Life Science, College of Natural Sciences, Chung-Ang University, Seoul, Korea; 3 Department of Anthropology and Archaeology, School of Social Science, National University of Mongolia, Ulaanbaatar, Mongolia; 4 Department of Archaeology and Art History, College of Humanities, Donga University, Busan, Korea; 5 Institute for Medical Sciences, College of Medicine, Chung-Ang University, Seoul, Korea; 6 Department of Laboratory Medicine, Chung-Ang University College of Medicine, Seoul, Korea; University of Florence, ITALY

## Abstract

Members of the Mongol imperial family (designated the Golden family) are buried in a secret necropolis; therefore, none of their burial grounds have been found. In 2004, we first discovered 5 graves belonging to the Golden family in Tavan Tolgoi, Eastern Mongolia. To define the genealogy of the 5 bodies and the kinship among them, SNP and/or STR profiles of mitochondria, autosomes, and Y chromosomes were analyzed. Four of the 5 bodies were determined to carry the mitochondrial DNA haplogroup D4, while the fifth carried haplogroup CZ, indicating that this individual had no kinship with the others. Meanwhile, Y-SNP and Y-STR profiles indicate that the males examined belonged to the R1b-M343 haplogroup. Thus, their East Asian D4 or CZ matrilineal and West Eurasian R1b-M343 patrilineal origins reveal genealogical admixture between Caucasoid and Mongoloid ethnic groups, despite a Mongoloid physical appearance. In addition, Y chromosomal and autosomal STR profiles revealed that the four D4-carrying bodies bore the relationship of either mother and three sons or four full siblings with almost the same probability. Moreover, the geographical distribution of R1b-M343-carrying modern-day individuals demonstrates that descendants of Tavan Tolgoi bodies today live mainly in Western Eurasia, with a high frequency in the territories of the past Mongol khanates. Here, we propose that Genghis Khan and his family carried Y-haplogroup R1b-M343, which is prevalent in West Eurasia, rather than the Y-haplogroup C3c-M48, which is prevalent in Asia and which is widely accepted to be present in the family members of Genghis Khan. Additionally, Tavan Tolgoi bodies may have been the product of marriages between the lineage of Genghis Khan’s Borjigin clan and the lineage of either the Ongud or Hongirad clans, indicating that these individuals were members of Genghis Khan’s immediate family or his close relatives.

## Introduction

Temujin was born into the Borjigin clan as a son of Yesugei, who was a grandson of Khabul (or Qabul) Khan (King in the Mongolian language), the first khan of the Khamag Mongol confederation. In 1206, Temujin annexed and unified many Mongol-Turkic nomadic tribes of Northeast Asia, and was then crowned the “Genghis Khan (the supreme king in the Mongolian language)” at a Kurultai, a general council of Mongol chiefs [1]. After founding the Mongol Empire, Genghis Khan invaded neighboring lands outward from the Mongolian plateau, ultimately conquering most of Eurasia. The Mongol Empire expanded to be the largest contiguous land empire in human history, covering the area from Eastern Europe to the East Sea/the Sea of Japan. The vast transcontinental empire allowed for the exchange of cultures and religions between Asia and Europe via the Silk Road. Thus, the Pax Mongolica greatly influenced many civilizations in Eurasia during the 13^th^ and 14^th^ centuries; indeed, its cultural, social, religious, and economic impact on the world remains today.

To solidify the foundation of the Mongol Empire, Genghis Khan employed two major strategies. First, because the Mongol Empire was too large to be controlled by a single ruler, he allocated the territories to his family members and let them rule their own independent territories [2, 3]. Because his wives were old and his sons were incompetent compared to his daughters, Genghis Khan bestowed upon his daughters, instead of his sons, the heavy responsibilities of shielding the inner territory of the Mongolian plateau and operating the outposts for his world conquest. He distributed the neighboring kingdoms surrounding the inner territory of the Mongolian plateau among his 4 daughters, including Alaqai Beki, “beki” meaning princess in the Mongolian language, who came to dominate the Ongud kingdom, Eastern Mongolia [2, 4]. His daughters faithfully ruled their own kingdoms throughout their lives on behalf of their father.

Genghis Khan’s second strategy was to use quda, the traditional marriage alliance system of Mongolia, to marry his sons and daughters into the ruling lineages of neighboring kingdoms such as the Ongud [3, 5]. Through this system, Genghis Khan expected his daughters to become regents of the kingdoms once dominated by their husbands (guregens; prince consorts in the Mongolian language); he forced guregens to go to war, leaving their wives (bekis) in charge of running the home according to Mongol tradition. In doing so, Genghis Khan conferred power to his daughters, and not to the guregens, to rule the kingdoms. Moreover, guregens could not return to their homeland for long periods of time, and were killed at a high rate in Genghis Khan’s war. Through these qudas, bekis, as authoritarian rulers, strengthened alliances among their kingdoms and provided Genghis Khan the solid foundation necessary to conquer many kingdoms outward from the Mongol steppe [2].

Thus, Genghis Khan could not have founded the Mongol Empire without his bekis dominating the kingdoms [2, 3]. Although Genghis Khan’s daughters wielded unprecedented political authority in several kingdoms of the Mongol Empire, their names and achievements in solidifying the Mongol Empire have disappeared from Mongol chronicles over generations. Their burial grounds have never been found because all members of the imperial family of the Mongol Empire, including khans, khatuns, meaning “empress” in Mongolian, bekis, and their descendants, were buried without identifying signs, according to the long-standing tradition of keeping the burial grounds of ancestors in a secret necropolis called “Lord’s Enclosure” [3–5]. Indeed, the geographical locations of the graves of the Mongol imperial family (designated the Golden family) members are unknown [1, 6, 7].

Many researchers believe that the discovery of graves of the ancients will undoubtedly reveal details of their genealogies and lives. Molecular archaeologists have developed scientific and systematic approaches to trace customs, diseases, and genealogies of the ancients as well as various activities during the lifetimes of ancient peoples. Accordingly, it is likely that direct molecular archaeological analysis of the human remains from Genghis Khan’s Golden family members who ruled Pax Mongolica will provide scientific clues to unveil their mysterious lives and genealogies.

In 2004, 7 graves were first excavated in the central hill of Tavan Tolgoi (“five hills” in the Mongolian language) by a Mongolian excavation team. Tavan Tolgoi lies within the Ongud province once dominated by Alaqai Beki and then Sorkhokhtani, a wife of Genghis Khan’s youngest son Tolui, during the early Mongolian era [8]. Mongolian archaeologists who participated in the excavation of Tavan Tolgoi graves strongly suspected that 5 of 7 Tavan Tolgoi graves belonged to the Golden family [9–11], and one of those 5 graves was thought to be that of a Mongol Queen. Burial artifacts excavated from the Tavan Tolgoi graves have since been displayed in the National Museum of Mongolian History, recognized as important relics of the Mongol Empire.

In this study, we aimed to determine the matrilineal and patrilineal origins of the Golden family members from the Tavan Tolgoi burial site and to determine kinship among them and with Genghis Khan, through analysis of single nucleotide polymorphism (SNP) and short tandem repeat (STR) of mitochondria, autosomes, and Y chromosomes from ancient DNAs (aDNAs). In addition, we compared their mitochondrial DNA (mtDNA) and Y-STR haplotypes with those of modern-day individuals using neighbor-joining (NJ) and Y-chromosome STR Haplotype Reference Database (YHRD) analyses, respectively, to determine the current geographical distribution of female- and male-line descendants of the Golden family members. Moreover, the familial relationship of the Tavan Tolgoi bodies with Genghis Khan was also postulated based on molecular archaeological and historical evidence. This study, the first molecular archaeological analysis of several skeletons belonging to Genghis Khan’s Golden family, presents molecular data to reveal the identity and genealogy of Golden family members, including a Mongol Queen, thereby unlocking the door to mysterious lives of the Golden family.

## Results

### Archaeological and physical anthropological analyses of the Tavan Tolgoi bodies

**Fig 1:**
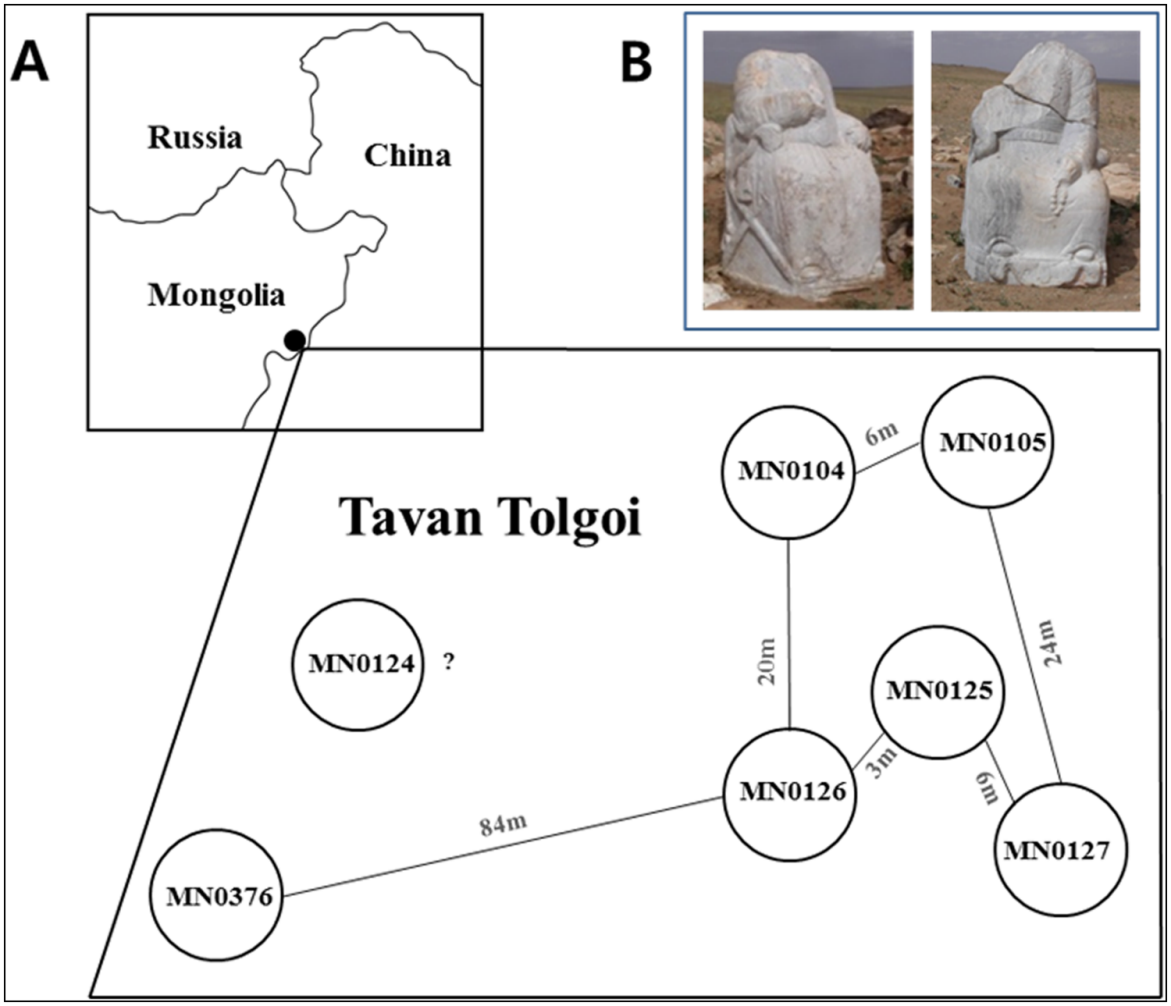
Geographical features of Tavan Tolgoi graves. A: A map of Tavan Tolgoi (top) and the relative geographical locations (bottom) of the graves excavated there. B: Two headless stone statues called Mongol King and Queen are located at the entrance of Tavan Tolgoi.

In 2004, a Mongolian excavation team from the Department of Anthropology and Archaeology, National University of Mongolia, discovered burial grounds scattered on the sunny slope of the center hill of five hills in Tavan Tolgoi. Tavan Tolgoi is geographically located near the Ongon district, Sukhbaatar province, Eastern Mongolia, 650 km away from the capital city of modern Mongolia, Ulan Bator (Fig 1A). It consists of a rocky hill slope amidst a huge plain that is 1,104 m above sea level and is located between Karakorum (present-day Kharkhorin), the capital city of the 13^th^ century Mongol Empire, and Dadu (present-day Beijing), the capital city of the Yuan Dynasty. Archaeological monuments, the remnants of several ancient nomadic tribes who had inhabited the region over thousands of years from the Neolithic to Mongolian eras, have been identified in this region. Out of them, two headless stone statues called “Mongolian King and Queen” by local people are regarded as very important relics, indicating that Tavan Tolgoi is a sacred region from the traditional Mongolian perspective (Fig 1B).

**Fig 2:**
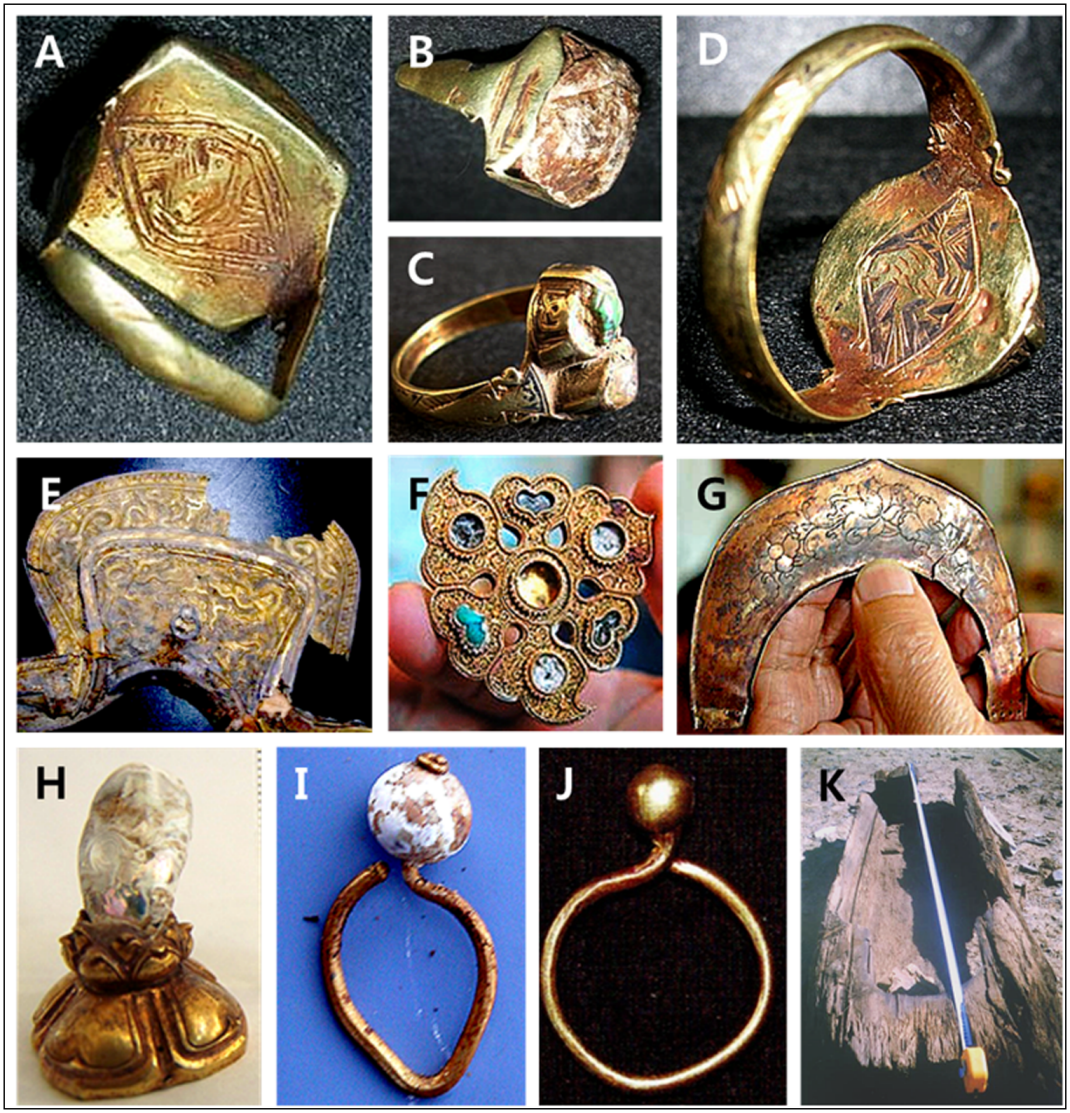
Some of the burial artifacts excavated from the Tavan Tolgoi graves. A-D: MN0105; A-B: a golden ring engraved with a falcon image, C-D: another golden ring engraved with the same falcon image as in A. E-G: MN0125; E: a saddle sheathed in gold dragon-shaped artistic decoration, F: the same golden ornament of boqta as those of the boqtas of Mongol khatuns in the design and shape, G: a golden ornament inside the boqta. H: Jins of MN0124. I: a golden earring of MN0126. J-K: MN0127; J: a golden earring, K: a coffin made of cinnamon.

The 7 Tavan Tolgoi graves had similar exterior surface structures surrounded by a ring-shaped stone construction with a diameter of 6–8 m, reflecting a tomb style typical of the Xiongnu era (from about the 3^rd^ century B.C. to the late 1^st^ century C.E.) [11, 12]. However, the internal structure of the Tavan Tolgoi graves and the style of burial artifacts, instead, indicated graves from the medieval Mongolian era (Fig 2 and S1 Table) [11, 13]. This was confirmed by Youn and colleagues [14], who used ^14^C radiocarbon dating of their human remains or artifacts to show that the Tavan Tolgoi graves dated between 1130–1250 AD. Mongolian archaeologists demonstrated that the surface features of the Tavan Tolgoi graves were probably intended to protect the graves from looting and enemies from the contemporary or future world, implying that these individuals were important figures in the society at that time [10, 11]. Thus, archaeological and radiocarbon dating results strongly suggest that the 7 Tavan Tolgoi graves correspond to the early Mongolian era, when Genghis Khan and his close family members, including his sons, daughters, sons-in-law, and daughters-in-law, were in power.

All physical anthropological parameters indicate that the skulls of the Tavan Tolgoi graves were all anthropologically Mongoloid (S2 Table). Unfortunately, not all of the cranial metric traits from the skulls of MN0125 and MN0127 were available because of significant breakage of the skeletons, including the skulls. The presumed height and weight of MN0104 and MN0105 were 169.8 cm and 78.1 kg and 165.6 cm and 68.1 kg, respectively. Particularly, MN0105 was more than 10 cm taller than other females of average height in the Mongolian era, indicating that she was well nourished and/or genetically superior [9, 15]. Estimation of height and weight failed in other Tavan Tolgoi bodies; the femurs of MN0124, MN0126, and MN0376 were badly broken and those of MN0125 and MN0127 were completely broken. In addition, their anatomical sex and presumed age at death, according to physical anthropological data obtained from osteometric and odontometric estimation of the skulls and/or pelvic bones, and teeth, respectively, are shown in Table 1 and S1 Table [16, 17]. Based on these data, it was estimated that the anatomical sex of MN0104, MN0126, MN0127, and MN0376 was male, while that of the other bodies (MN0105, MN0124, and MN0125) was female. The results of physical anthropological analysis were coincided with those of amelogenin sex determination by the conventional PCR analysis using X-Y primers. One Tavan Tolgoi body (MN0124) was estimated to have died in her 10s, 4 (MN0104, MN0125, MN0126, and MN0127) in their 20s, and 2 (MN0105 and MN0376) in their 40s or 50s.

**Table 1:** mtDNA haplogroups of the Tavan Tolgoi bodies.

Sample	Sex Determination		mtDNA^c^			Hp
	Anth^a^	Amel^b^	HVR1 (15977–16399)	HVR2 (29–381)	Coding Region	
***First Laboratory***						
MN0104^d^	male	XY	16171G 16223T 16311C 16362C	73G 263G 309+C 310+C	3010A, 5178A	D4
MN0105^e^	female	XX	16093C 16223T 16261T 16288C 16298C	73G 249del 263G 309+C 310+3C	10398G, 4715G	CZ
MN0125^f^	female	XX	16171G 16223T 16311C 16362C	73G 263G 309+C 310+C	3010A, 5178A	D4
MN0126^g^	male	XY	16171G 16223T 16311C 16362C	73G 263G 309+C 310+C	3010A, 5178A	D4
MN0127^h^	male	ND	16171G 16223T 16311C 16362C	73G 263G 309+C 310+C	3010A, 5178A	D4
MN0124^i^	female	XX	16217C	73G 152C 195C 263G 309+C 310+C	10398C, 12705C	R
MN0376^j^	male	XY	16223T 16234T 16299G 16362C	73G 146C 217C 263G 309+C 310+C	10398G, 3394C	M9
***Second Laboratory***						
MN0104	male	XY	16171G 16223T 16311C 16362C	73G 263G 309+C 310+C	3010A, 5178A	D4
MN0105	female	XX	16093C 16223T 16261T 16288C 16298C	73G 249del 263G 309+C 310+3C	10398G, 4715G	CZ
MN0125	female	XX	16171G 16223T 16311C 16362C	73G 263G 309+C 310+C	3010A, 5178A	D4
MN0126	male	XY	16171G 16223T 16311C 16362C	73G 263G 309+C 310+C	3010A, 5178A	D4
MN0127	male	ND	16171G 16223T 16311C 16362C	73G 263G 309+C 310+C	3010A, 5178A	D4
MN0124	female	XX	16217C	73G 152C 195C 263G 309+C 310+C	10398C, 12705C	R
MN0376	male	XY	16223T 16234T 16299G 16362C	73G 146C 217C 263G 309+C 310+C	10398G, 3394C	M9
***Researchers***						
Lab Worker 1		XX	16223T 16362C	73G 195C 198T 263G 315+C		D4/G
Lab Worker 2		XX	16172C 16304C 16362C	73G 263G 309+C 315+C		R9b
Archaeologist 1		XX	16092C 16129A 16223T 16266T 16271C	73G 263G 291T 309+C 310+C		-
Archaeologist 2		XX	16189C 16223T 16257A 16261T 16269G	73G 150T 263G 309+C 310+C		-
Archaeologist 3		XY	16092C 16129A 16223T 16271T 16262C	73G 150T 263G 290T 309+C 310+C		-

^a^Anthropological sex identification

^b^Sex identification by means of PCR analysis of amelogenin

^c^Mutation sites that were identified by comparing the Tavan Tolgoi bodies with rCRS in the control regions (HVR1 and HVR2) and the coding regions are indicated by the nucleotide positions of the mutated bases in their mtDNA

^d^GenBank accession number: KF898382 and KF926840 for HVR1 and HVR2 respectively

^e^GenBank accession number: KF898383 and KF926841 for HVR1 and HVR2 respectively

^f^GenBank accession number: KF898385 and KF926843 for HVR1 and HVR2 respectively

^g^GenBank accession number: KF898386 and KF926844 for HVR1 and HVR2 respectively

^h^GenBank accession number: KF898387 and KF926845 for HVR1 and HVR2 respectively

^i^GenBank accession number: KF898384 and KF926842 for HVR1 and HVR2 respectively

^j^GenBank accession number: KF898388 and KF926846 for HVR1 and HVR2 respectively.

Letters (A, C, G, and T), del and (+) after specific nucleotide positions mean a substitution, deletion and insertion, respectively, of the indicated bases. Minus (-) indicates that mtDNA was not assigned to a specific haplogroup. ND: not determined, and Hp: haplogroup.

Burial artifacts from the 5 Tavan Tolgoi graves were exceptional in quality and quantity, compared to those previously found in other graves from the Mongolian era; most artifacts from the Tavan Tolgoi graves, however, including MN0104, MN0126, and MN0127, had been looted a long time ago [9]. In the female graves, MN0105 and MN0125, we unearthed golden rings engraved with the falcon image that symbolizes the Genghis Khan and the Borjigin clan, a saddle sheathed in gold dragon-shaped artistic decoration and golden ornaments for boqta, which are similar to those of Mongol khatuns in shape and decoration (Fig 2A–2G). In the male graves, MN0104 was unearthed holding an ornament, called “Jins” in Mongolia, consisting of a large pearl mounted on a flower-shaped golden base (Fig 2H). MN0126 and MN0127 were each wearing only a single earring that was found under the skull, indicating their higher social status (Fig 2I and 2J); Mongolian aristocratic men usually wore hats adorned with Jins and a single earring in their left ears, confirming that MN0104, MN0126 and MN0127 were all males. In addition, the coffin wood of MN0127 was not from trees native to the Mongolian Plateau; the wood was cinnamon (*Cinnamomum sp*.), which grows only in hot and humid Southeast Asia, at least several thousand kilometers away from Tavan Tolgoi. This finding suggests that he and his family had considerable financial and political power sufficient to bring the plant for his coffin from a far-off province. Taken together, these data clearly indicate that the 5 graves belong to the Golden family members who were contemporaries of Genghis Khan, including a Mongol Queen (MN0125), although their exact identities are ambiguous [9–11].

On the contrary, the internal structure and lack of notable artifacts, aside from a pair of small earrings, in the MN0124 grave containing the teenage female skeleton indicate that it belonged to a member of the general public of the Mongolian era. MN0376 was also believed to belong to a member of the general public, based on the artifacts excavated. The burial artifacts from this grave were all associated with war, including an arrow quiver and arrowheads; his physical anthropological characteristics, including several signs of injury during his lifetime, such as trauma of the left clavicle and a well-developed skeleton, indicate that he was a common warrior [9, 11].

### Authenticity assessment of aDNA analysis

To prevent cross-contamination of DNA samples, bone samples were collected with extensive precautions according to previously published procedures [18–20]. We ensured that our data were derived from genuine aDNAs, and not from contaminated DNA samples, based on the following precautions. First, our data were obtained from at least 6 independent multiple extractions and amplifications per sample. In detail, aDNAs from Tavan Tolgoi bodies were independently extracted more than 3 times per sample in two independent laboratories, and then their haplotypes and haplogroups of mtDNA and Y chromosome were determined by independent PCR amplification more than two times per extract. DNA sequences obtained from at least 6 (3 extractions per sample × 2 PCR amplifications per extract) independent experiments per sample were compared to determine the similarity among the obtained nucleotide sequences, and the DNA sequences were confirmed to be identical in at least 5–6 experiments and were regarded as a consensus sequence for the specific sample. Second, our data were obtained from 2 independent laboratories located in different buildings and managed by different principal investigators; if the data obtained were not identical, all procedures were repeated beginning with the extraction step of aDNA. Third, we compared mtDNA data from aDNAs with these of all researchers who participated in the archaeological excavation, aDNA extraction, and PCR amplification of aDNA (Table 1); if the aDNA and modern DNA sequences matched, these samples were excluded from the analysis. Fourth, to support that our PCR products were derived from human aDNA, sequences of hypervariable region 1 (HVR1) of horse mtDNA were amplified using previously reported primer sets (S1 Fig). Ancient horse bones excavated together with MN0105 were subjected to mtDNA analysis. The D-loop HVR1 of horse mtDNA was successfully amplified and sequenced with no evidence of contamination by human aDNA. In addition, the DNA sequences of amplified horse aDNA were compared to human DNA sequences from the Tavan Tolgoi bodies and from researchers of the 2 laboratories, as well as to the revised Cambridge Reference Sequence (rCRS), using SeqMan II software [18, 21]. The results demonstrate that our data were sufficiently reproducible for selective analysis of aDNA from ancient human skeletons and were not contaminated by organelles or contemporary individuals. Fifth, uracil DNA glycosylase (UDG) treatment of purified aDNA reduces the risk of identifying incorrect DNA sequences by inhibiting the erroneous PCR amplifications and deamination of deoxycytidine residues in the aDNA template. Therefore, we compared DNA sequences obtained from direct PCR amplification with those from clones constructed after treating aDNA samples of 6 Tavan Tolgoi bodies, except for MN0127, with either active or inactive UDG (S3 and S4 Tables). The consensus nucleotide sequences from the direct PCR and the clones were identical, including the sites of haplogroup-defining SNPs, indicating that all data obtained from the 6 Tavan Tolgoi bodies were indeed from aDNA and did not contain significant base modifications. One exception was found at nucleotide position 16,250 of HVR1 in MN0376, which showed a transition from C to T when the clones were inserted by MN0376 aDNA treated with inactive UDG (S4 Table). However, the reversion of transition from T to C was observed when clones were inserted with MN0376 aDNA treated with active UDG, indicating that UDG effectively inhibits post-mortem DNA damage.

### Identification of mtDNA haplogroups and haplotypes of the Tavan Tolgoi bodies and their matrilineal origins

Skeletons of 7 Tavan Tolgoi graves were analyzed for haplotypes and haplogroups of their mtDNAs through DNA sequencing of control regions, HVR1 and HVR2, and several coding regions. Their mtDNA haplotypes were determined by comparison of HVR1 and HVR2 DNA sequences with rCRS; mtDNA haplogroups were assigned by DNA sequence analysis of 10 additional coding regions and the control regions, HVR1 and HVR2, for obtaining unambiguous results [18, 22–25]. PCR amplification and DNA sequencing of the control and coding regions were successfully performed in all bodies examined by using primer sets presented in S5 Table; results obtained from the two laboratories were identical.

Overall, 4 haplotypes were identified in the 7 Tavan Tolgoi bodies and were assigned to 4 haplogroups (Table 1). Four bodies, MN0104, MN0125, MN0126, and MN0127, likely members of the Golden family according to archaeological and physical anthropological analyses, were identical in their mtDNA sequences and were assigned to the same haplogroup, D4, prevalent in Far Eastern Asia [26, 27]. Meanwhile, the other Golden family member, MN0105, was assigned to the CZ haplogroup, which is prevalent in Northeastern Asia (mostly in Siberia), implying that MN0105 had no kinship with the other Golden family members of Tavan Tolgoi. The haplogroups of the members of the general public, MN0124 and MN0376, were R and M9, occurring mostly in Arabian plate and South East Asia including Tibet, respectively [28–30].

### Identification of Y-haplogroups and haplotypes of the Tavan Tolgoi bodies and their patrilineal origins

Patrilineal origins of Tavan Tolgoi bodies were first traced by Y-haplogrouping using 10 biallelic Y chromosome markers. Four (MN0104, MN0126, MN0127 and MN0376) of 7 Tavan Tologoi bodies were determined to be males based on physical anthropological estimation and molecular sex determination using amelogenin analysis (Table 1 and S1 Table). Unfortunately, MN0127, who was believed to belong to the Golden family, could not undergo Y-haplogroup analysis because the amount of DNA purified was insufficient for PCR amplification of the Y chromosome, which exists as only one copy within a cell.

MN0104 was positive only for R-M207 and was negative for O-M175, C-RPS4Y, N-M231, D-M174, J-M304, and Q-M242 (Table 2 and S2 Fig). In addition, MN0104 carried the R1-defining M173 and R1b-defining M343 biallelic markers, but not R1a1a-defining M17. MN0126 was also positive for the same biallelic markers (R-M207 and R1b-M343) as those carried by MN0104 and negative for O-M175 and C-RPS4Y (Table 2 and S3 Fig) [31, 32]. These data suggest that male members, MN0104 and MN0126, of the Golden family belong to haplogroup R1b-M343; however, only 4 biallelic markers were definable in MN0126 [31]. In contrast, the Mongolian warrior MN0376 was positive for R-M207 and negative for O-M175, C-RPS4Y, N-M231, D-M174, J-M304, and Q-M242. MN0376 was affiliated with R1a1a-M17, a subclade of haplogroup R1, rather than R1b-M343 because he carried R-M207, R1-M173, and R1a1a-M17 but not R1b-M343 (Table 2 and S4 Fig) [31, 32].

**Table 2:** Y-haplogroups of the male Tavan Tolgoi bodies.

Sample	M175	RPS4Y	M231	M174	M304	M242	M207^a^	M173^b^	M17^c^	M343^d^	Hp
	(O)	(C)	(N)	(D)	(J)	(Q)	(R)	(R1)	(R1a1a)	(R1b)	
	5-bp del	C→T	G-A	T→C	T→G	C→T	A→G	A→C	G del	C→A	
MN0104	TTCTC	C	G	T	T	C	G	C	G	A	R1b
MN0126	TTCTC	-	-	-	T	C	G	-	-	A	R1b
MN0376	TTCTC	C	G	T	T	C	G	C	G del	ND	R1a1a

^a^GenBank accession number: KJ003992, KJ003993 and KJ003994 for MN0104, MN0126 and MN0376 respectively

^b^GenBank accession number: KJ003995 and KJ003996 for MN0104 and MN0376 respectively

^c^GenBank accession number: KJ003997 for MN0376

^d^GenBank accession number: KJ003998 and KJ003999 for MN0104 and MN0126 respectively.

Minus (-) and del indicate failure of PCR amplification and deletion of the indicated bases, respectively. Letters (A, C, G, and T) in the data indicate the bases in the haplogroup-defining sites. Hp: haplogroup, and ND: not done.

To ascertain whether the R1b-M343-carrying males shared an identical haplotype, we determined their Y-allelotypes using 16 Y-STR markers (Table 3 and S5 Fig). Y-STR markers were examined in bodies MN0104, MN0126, and MN0376, but not MN0127, again due to PCR failure. Y-STR haplotypes of MN0104 and MN0376 were successfully identified in all of 16 marker loci examined, whereas only 8of 16 marker loci were determined in MN0126. Two Golden family members, MN0104 and MN0126, contained the consensus Y-STR allelotypes, which were identical in 7 of 8 definable loci and shared 1 of 2 alleles in a DYS385 marker locus. In contrast, 10 of the 16 marker loci that were definable were not identical in Y-STR allelotypes between MN0104 and MN0376, and 5 of 8 loci between MN0126 and MN0376 did not match in their Y-STR allelotypes. These results are fully consistent with the Y-SNP results, indicating that MN0104 and MN0126 had identical Y-haplotypes from the same patrilineal origin, whereas MN0376 shared no patrilineal origin with the two male members of the Golden family.

**Table 3:** Y-STR allelic profiles of the male Tavan Tolgoi bodies.

Sample	Hp	Y-STR Marker															
		DYS19	DYS385	DYS389I	DYS389II	DYS390	DYS391	DYS392	DYS393	DYS437	DYS438	DYS439	DYS448	DYS456	DYS458	DYS635	YGATAH4
MN0104	R1b	14	13/17	13	30	22	11	13	13	15	10	12	20	15	15	23	11
	R1b	14	13/17	13	30	22	11	13	13	15	10	12	20	15	15	23	11
	R1b	14	13/17	13	30	22	11	13	13	15	10	12	20	15	15	23	11
	**Consensus**	**14**	**13/17**	**13**	**30**	**22**	**11**	**13**	**13**	**15**	**10**	**12**	**20**	**15**	**15**	**23**	**11**
MN0126	R1b	14	13	-	-	-	11	-	13	-	-	12	-	-	15	23	11
	R1b	14	13	-	-	-	11	-	13	-	-	12	-	-	15	23	11
	R1b	14	13	-	-	-	11	-	13	-	-	12	-	-	15	23	11
	**Consensus**	**14**	**13**	**-**	**-**	**-**	**11**	**-**	**13**	**-**	**-**	**12**	**-**	**-**	**15**	**23**	**11**
MN0376	R1a1a	16	11/14	13	29	26	11	11	13	14	11	10	20	15	17	23	8/13
	R1a1a	16	11/14	13	29	26	11	11	13	14	11	10	20	15	17	23	13
	R1a1a	16	11/14	13	29	26	11	11	13	14	11	10	20	15	17	23	13
	**Consensus**	**16**	**11/14**	**13**	**29**	**26**	**11**	**11**	**13**	**14**	**11**	**10**	**20**	**15**	**17**	**23**	**13**

Consensus Y-STR profiles are indicated in bold characters. Minus (-) indicates failure of PCR amplification. Hp: haplogroup.

In addition, the Y-STR profiles from YHRD were screened to identify individuals with the same STR profile as that of MN0104. PowerPlex Y and Yfiler of YHRD, which enable comparison of a total of 11 and 16 Y-STR marker loci among samples, respectively, were compared with the Y-STR profile of MN0104 with Y-STR profiles from YHRD (S6 Table). Two individuals who perfectly matched MN0104 in all 16 Y-STR definable marker loci were identified as 1 Kalmyk (named Kalmyk 73) and Hui (Chinese) through Yfiler of YHRD. One individual, Uzbek (unnamed), was found to match MN0104 in all 11 Y-STR marker loci through PowerPlex Y of YHRD. Moreover, the Y-STR profiles of the 3 individuals (Hui, Kalmyk, and Uzbek) were applied to determine their Y-haplogroups using Yfiler or PowerPlex Y of YHRD; these individuals were not previously assigned to specific Y-haplogroups in the literature. They were all assigned to the same Y-haplogroup R1b as MN0104 with the highest probability, suggesting that male Golden family bodies carried the Y-haplogroup R1b.

Additionally, individuals with the Y-STR profile matching that of MN0104 in 16 definable marker loci were searched in the literature reporting Y-STR profiles associated with modern Eurasian populations (S6 Table) [33–35]. Two Russian (named Kalmyk 73 and Russian), 1 Uzbek (named 26), and 1 Tajik (named 134) individuals were found to match MN0104 in 14–16 definable Y-STR marker loci. One (named Kalmyk 73) of 2 Russians was confirmed to be the same individual as the Kalmyk described above and searched from YHRD. The other 3 individuals including Russian, Uzbek, and Tajik were previously assigned to the Y-haplogroups R1*-M173, R1b1a*-P297, and R1b1a*-P297, respectively, by Y-SNP analysis [34, 35], except for Kalmyk 73 which was not previously assigned to the specific Y-haplogroup in the literature [33]. The Russian matched MN0104 in 15 of 16 marker loci, exhibiting a different allele from MN0104 in the DYS439 Y-STR marker locus. Two other individuals (Uzbek and Tajik) whose Y-STR profiles were identical in all 16 marker loci did not match MN0104 in 2 marker loci (DYS389II and DYS458). MN0104, Kalmyk, and the other Russian (named Russian) were also assigned to the Y- haplogroup R1b through Yfiler of YHRD, while the other individuals, Uzbek (named 26) and Tajik, were not assigned to the specific Y-haplogroup through Yfiler of YHRD.

In contrast, individuals with the same Y-STR profiles as that of MN0376 were screened through Yfiler and PowerPlex Y from YHRD and the literature (S7 Table). Individuals matching MN0376 in the Y-STR profiles defined through Yfiler of YHRD were not identified, whereas 8 individuals perfectly matched MN0376 in all 11 definable Y-STR marker loci through PowerPlex Y of YHRD. In addition, none were previously assigned to the specific Y-haplogroup based on Y-SNP analysis and were affiliated with a specific Y-haplogroup through PowerPlex Y of YHRD. However, 7 individuals who matched MN0376 in 13 of 16 marker loci were identified in the literature [34–38], including 1 Russian, 1 Hui (Chinese), 1 Croatian, and 4 Pashtun. Two individuals (Russian and Pashtun) were previously assigned to the Y-haplogroup R1a1a-M17 using Y-SNP analysis, and the 3 other Pashtuns were assigned to the Y-haplogroup R1a1a*-M198 (S7 Table). The Hui (Chinese) and Croatian were not previously assigned to the specific Y-haplogroup using Y-SNP analysis. All 7 individuals were affiliated with the Y-haplogroup R1a through Yfiler of YHRD, whereas MN0376 was not assigned to a specific Y-haplogroup.

Taking all data of Y-SNP and Y-STR into consideration, the male Golden family bodies (MN0104, MN0126, and MN0127) from Tavan Tolgoi were clearly assigned to the Y-haplogroup R1b with the identical Y-haplotype, whereas a male general public (MN0376) to the Y-haplogroup R1a1a showed a quite different Y-haplotype from that of male members of the Golden family.

### Determination of autosomal STR (A-STR) allelotypes of the Tavan Tolgoi bodies

To define the exact kinship among 4 Golden family members (MN0104, MN0125, MN0126 and MN0127) who shared the same SNP and/or STR profiles of mtDNA and Y chromosome each other, their A-STR allelic profiles were further examined using a total of 9 A-STR markers, including amelogenin for sex determination, and estimated by GeneMapper software (Table 4). STR profiles of autosomal DNA and amelogenin were reproducible for 6 Tavan Tolgoi bodies, except for MN0127 due to failed PCR amplification. Consensus A-STR profiles were successfully determined for the 6 bodies based on results obtained from at least a total of 6 independent experiments carried out in 2 independent laboratories, except that MN0126 was not clearly determined for the consensus allelotypes in a D21S11 marker locus, showing 30/30 or 30/33.2.

**Table 4:** A-STR allelic profiles of the Tavan Tolgoi bodies.

Sample^a^	DNA Extraction Repeat	Amel^b^	D13S317	D7S820	D2S1338	D21S11	D16S359	D18S51	CSF1PO	FGA
**MN0104**	1 (Lab. 1)	XY	9/11	10/12	17/20	29/29	9/12	12/13	11/12	21/27
	2 (Lab. 1)	XY	9/11	10/12	17/20	29/29	9/12	12/13	11/12	21/27
	3 (Lab. 2)	XY	9/11	10/12	17/20	29/29	9/12	12/13	12/12	21/27
	**Consensus**	**XY**	**9/11**	**10/12**	**17/20**	**29/29**	**9/12**	**12/13**	**11/12**	**21/27**
**MN0105**	1 (Lab. 1)	XX	8/11	11/12	23/23	29/32.2	9/12	14/19	11/12	24/26
	2 (Lab. 1)	XX	8/11	11/12	23/23	29/29	9/12	14/19	11/12	24/26
	3 (Lab. 2)	XX	8/11	11/12	23/23	29/32.2	9/12	14/19	11/12	24/26
	**Consensus**	XX	**8/11**	**11/12**	**23/23**	**29/32.2**	**9/12**	**14/19**	**11/12**	**24/26**
**MN0125**	1 (Lab. 1)	XX	8/9	10/12	17/20	29/33.2	9/12	12/13	11/12	19/27
	2 (Lab. 1)	XX	8/9	10/12	17/20	29/33.2	9/12	12/13	11/12	19/27
	3 (Lab. 2)	XX	8/9	10/12	17/20	29/33.2	9/12	12/13	11/12	19/27
	**Consensus**	**XX**	**8/9**	**10/12**	**17/20**	**29/33.2**	**9/12**	**12/13**	**11/12**	**19/27**
**MN0126**	1 (Lab. 1)	XY	8/9	12/12	20/24	30/30	9/11	12/16	11/12	19/22
	2 (Lab. 1)	-	-	-	-	-	-	-	-	-
	3 (Lab. 2)	XY	8/9	12/12	20/24	30/33.2	9/11	12/16	11/12	19/22
	**Consensus**	**XY**	**8/9**	**12/12**	**20/24**	**30/30 or 30/33.2**	**9/11**	**12/16**	**11/12**	**19/22**
**MN0124**	1 (Lab. 1)	XX	11/12	8/8	17/23	29/30	9/12	13/13	12/13	23/24
	2 (Lab. 1)	XX	11/12	8/8	17/23	29/30	9/9	13/16	12/13	23/24
	3 (Lab. 2)	XX	11/12	8/8	17/23	29/30	9/12	13/16	12/13	23/24
	**Consensus**	**XX**	**11/12**	**8/8**	**17/23**	**29/30**	**9/12**	**13/16**	**12/13**	**23/24**
**MN0376**	1 (Lab. 1)	XY	12/12	10/10	23/27	31/32.2	9/9	13/24	9/12	21/23
	2 (Lab. 1)	XY	12/12	10/10	23/27	31/32.2	9/9	13/24	9/12	21/23
	3 (Lab. 2)	XY	12/12	10/10	23/27	31/32.2	9/9	13/24	9/12	21/23
	**Consensus**	**XY**	**12/12**	**10/10**	**23/27**	**31/32.2**	**9/9**	**13/24**	**9/12**	**21/23**

^a^Data of MN0127 were omitted from the table because an A-STR profile of MN0127 could not be determined because of failure of PCR amplification.

^b^Sex identification using PCR amplification of amelogenin.

All data were obtained using AmpFlSTR^®^ MiniFiler^™^ PCR Amplification Kit (Applied Biosystems Inc.). Consensus A-STR profiles are indicated in bold characters. Minus (-) indicates failure of PCR amplification.

The results of amelogenin-based sex identification in the Tavan Tolgoi bodies were consistent with physical anthropological analysis, confirming the validity of our A-STR profiling (Tables 1 and 4). The A-STR profiles of the 6 bodies were defined by allelotypes for 8 loci as indicated in Table 4. Inconsistency among consensus A-STR profiles was found in 5 of 48 total consensus allelotypes detected in all 8 marker loci in 6 Tavan Tolgoi bodies. Despite these exceptions, all other A-STR profiles were perfectly consistent in the 43 consensus allelotypes. MN0125 shared at least one allele with either MN0104 or MN0126 in all 8 marker loci examined except for amelogenin. Nevertheless, MN0104 and MN0126 did not share both alleles in 2 marker loci (D21S11 and FGA), but they shared at least 1 of 2 alleles in the remaining 6 marker loci. On the contrary, other Tavan Tolgoi bodies (MN0105, MN0124, and MN0376) shared no alleles in 2 or more loci with the 3 Golden family members (MN0104, MN0125, and MN0126) and among themselves. These results strongly indicate that MN0104, MN0125 and MN0126 have some family ties, but no family relationship with other Tavan Tolgoi bodies (MN0105, MN0124 and Mn0376), although the exact kinship among them is ambiguous.

### Present geographical distribution of modern-day individuals with the same mtDNA haplotypes or Y-STR haplotypes as those of Tavan Tolgoi bodies

To assess the present geographical distribution of the same mtDNA haplotypes found in the Tavan Tolgoi bodies, HVR1 and HVR2 DNA sequences were compared with those of 8,478 modern-day individuals from GenBank database, and were used to construct NJ trees using MEGA version 6.0.6 [18, 24, 39]. The 4 D4-carrying bodies (MN0104, MN0125, MN0126, and MN0127) were assigned to categories corresponding to modern Eastern Asians such as Japanese, Chinese and Mongolian in NJ trees, exhibiting 98–99% similarity in their mtDNA sequences spanning a total of 664–673 base pairs of HVR1 and HVR2 (Table 5 and S6 Fig). Meanwhile, the CZ-carrying body (MN0105) had a close genetic relationship with modern Northeastern Asians such as the Yukaghir, Yakut, Xibo, Szekely and Hezhen (Table 5 and S7 Fig). The R-carrying teenager (MN0124) exhibited high sequence similarity with East Europeans and Middle Eastern and South Asians, including populations of Slavs, Druze, Indians and Slovakians (Table 5 and S8 Fig). The M9-carrying warrior (MN0376) exhibited high sequence similarity with South East Asians including Indonesians and Taiwanese (Table 5 and S9 Fig).

**Table 5:** Geographic distribution of the modern-day individuals with the same mtDNA haplotypes as those of the Tavan Tolgoi bodies.

Sample	mtDNA Haplogroup	Haplotype of HVR1 and HVR2^a^	Population^b^
MN0104/MN0125/MN0126/MN0127	D4	16171G 16223T 16311C 16362C 73G 263G 309+C 310+C	5 Japanese, 3 Chinese, 2 Mongolian, 1 Indonesian, 1 Russian, 1 Ethiopian, 1 Papua New Guinean
MN0105	CZ	16093C 16223T 16261T 16288C 16298C 73G 249del 263G 309+C 310+3C	5 Yukaghir, 4 Yakut, 3 Xibo, 3 Szekely, 2 Hezhen, 1 Chinese, 1 Japanese, 1 Greek, 1 Khanty, 1 Koryak, 1 Tuva, 1 Ulchi, 1 Mongolian, 1 Nganasan, 1 Tubalar
MN0124	R	16217C 73G 152C 195C 263G 309+C 310+C	5 Slav, 4 Druze, 2 Indian, 2 Slovakian, 1 Kchanti, 1 Hispanic, 1 Russian, 1 Xibo, 1 Papago, 1 Czeck
MN0376	M9	16223T 16234T 16299G 16362C 73G 146C 217C 263G 309+C 310+C	4 Indonesian, 3 South East Asian, 2 Asian, 1 Japanese, 1 Kazakh, 1 Tsou, 1 Hezhen, 1 Palestine, 1 Puyuma, 1 Saisiat, 1 Yakut, 1 Yukaghir

^a^Letters (C, G, and T), del and (+) after the specific nucleotide positions mean a substitution, deletion and insertion, respectively, of the indicated bases.

^b^Data were obtained by comparing the mtDNA haplotypes of the Tavan Tolgoi bodies with those of 8,478 modern-day individuals using the neighbor-joining analysis of concatenated HVR1 and HVR2.

MN0104, a male member of the Golden family, was tested using Yfiler and PowerPlex Y of YHRD to assess the geographical distribution of individuals with the same Y-STR profiles as the Tavan Tolgoi (S6 Table). A total of 6 modern-day individuals who matched MN0104 in at least 14 of 16 Y-STR marker loci were identified through YHRD and the literature. These individuals were mainly distributed in East Asia and Central Asia, including the territories (China, Kalmykia, Russia, Uzbekistan, and Tajikistan) associated with the past Mongol khanates.

By comparison, the Y-STR profile of MN0376 perfectly matched those of 8 individuals distributed across Eurasia including India, China, Pakistan, the Czech Republic, and Poland when 11 Y-STR marker loci were considered for matching MN0376 with individuals searched through the PowerPlex Y of YHRD (S7 Table). In addition, when we further examined published literature related to Y-STR profiles associated with modern Eurasian populations and compared the obtained data with the Y-STR profile of MN0376, 7 individuals who matched MN0376 in 13 of 16 definable Y-STR marker loci were identified. These individuals were distributed mainly in West Eurasia, including Russia, Croatia, Pashtun, and China.

Collectively, the results of mtDNA, Y-SNP, and Y-STR indicate that the Golden family members from Tavan Tolgoi had matrilineal (D4 and CZ) and patrilineal (R1b-M343) lines with genealogical origins in East Asia and West Eurasia, respectively. In addition, the Y-STR data were consistent with those of Y-SNP, demonstrating that male members of the Golden family, which showed identical Y-STR profiles, are affiliated with the Y-haplogroup R1b-M343 and that their direct descendants are distributed in West Eurasia from Poland to the western region of China.

## Discussion

### Golden family members of Tavan Tolgoi reveal the genealogical admixture between Caucasoid and Mongoloid ethnic groups

So far, no molecular archaeological study of members of the Mongolian imperial family has been conducted; this is largely because no grave of imperial family, especially those of the Golden family, has been identified until the Tavan Tolgoi grave excavation. To the best of our knowledge, this study is the first molecular archeological attempt to define the genealogy of Genghis Khan’s Golden family members in the Mongolian era.

Evidence suggests that many Mongoloid and Caucasoid nomadic tribes inhabited the present-day Mongolian plateau over thousands of years [40]. Since Genghis Khan’s era, the Mongolian population underwent rapid and considerable gene flow from Eurasia, resulting in additional genetic admixture [40]. Likewise, the Mongolian population was formed by the continuous admixture of indigenous tribes who inhabited the Mongolian plateau, with European and other Asian populations who inhabited regions geographically distant from Mongolia. This admixture has deeply influenced the physical appearance of modern-day Mongolian people, exhibiting both Mongoloid and Caucasoid features.

The mixing between Mongoloid and Caucasoid ethnic groups inherent in the genetic structure of modern-day Mongolians was also observed in the Tavan Tolgoi bodies. The Golden family members carried mtDNA haplogroups D4 and CZ, mostly found in Far Eastern and Northeastern Asia, respectively, whereas male members of Golden family carried the Y-haplogroup R1b-M343, dominant in Western Europe [41–43]. That is, although members of Golden family were physically Mongoloid, their molecular genealogy revealed the admixture between Caucasoid and Mongoloid ethnic groups. Thus, it is likely that their Mongoloid appearance would have resulted from gradual changes in their appearance from Caucasoid to Mongoloid through generations from their ancestors. Their physical appearance was largely attributed to D4-carrying Mongoloid females who were indigenous peoples of the Mongolian plateau, rather than an R1b-M343-carrying Caucasoid male spouse who had initially moved from Europe to the Mongolian plateau and his male descendants; it is, however, uncertain how and when the admixture between Mongoloid and Caucasoid ethnic groups originated in the Mongolian plateau.

### Y-haplogroup R1b-M343 of Tavan Tolgoi bodies may be another candidate for the Y-haplogroup of Genghis Khan and Genghis Khan’s Borjigin clan

Although many regard the portrait at the National Palace Museum in Taipei, Taiwan, as the depiction most closely resembles Genghis Khan, all existing portraits, including this one, are essentially arbitrary interpretations of Genghis Khan’s appearance by historians living generations after Genghis Khan’s era [2, 6]. Although the factual nature of the statement is controversial, Persian historian Rashid-al-Din reported in his “Jami’s al-tawarikh” written at the start of the 14^th^ century that most Borjigin ancestors of Genghis Khan were tall, long-bearded, red-haired, and bluish green-eyed, suggesting that the Genghis Khan’s male lineage had some Caucasoid-specific genetic features [44]. He also said that Genghis Khan looked just like his ancestors, but Kublai Khan, his grandson, did not inherit his ancestor’s red hair, implying that the addition of Mongoloid-specific alleles for determining hair color to the genetic makeup of Genghis Khan’s Borjigin clan was probably from the grandmother or mother of Kublai Khan, that is, the wife or daughter-in-law of Genghis Khan.

“On” and “gud” from Ongud mean West and plural in ancient Altaic language, respectively, implying that the Ongud is a tribe from Western Asia. In fact, the ancestors of the Ongud are the Shato Turks of the Western Göktürks Khaganate [45, 46]; they moved to Eastern Xinjiang in the 7^th^ century, and were scattered over Northern China and Inner Mongolia in the 9^th^ century [47]. In the Mongolian era, many Ongud peoples were resettled in Khorazm of Western Central Asia, as governors for the Golden Horde Dynasty, and eventually formed part of the Kazakhs and the Mughals [44]. In addition, they also fell under the Chagatai Khanate that was ruled by Chagatai Khan and his descendants and/or successors and extended from the Southern part of the Aral Sea to the Altai Mountains [48]. These suggest the possibility that the Ongud clan may be anthropologically Caucasoid rather than Mongoloid, according to their geographical origin. Therefore, the male bodies carrying R1b-M343 (prevalent in Western Europe) from Tavan Tolgoi, which was located within the territory of the Ongud Kingdom during the early Mongolian era, could be related to the Ongud male lineage, implying that Tavan Tolgoi bodies are genealogically Caucasoid.

**Fig 3:**
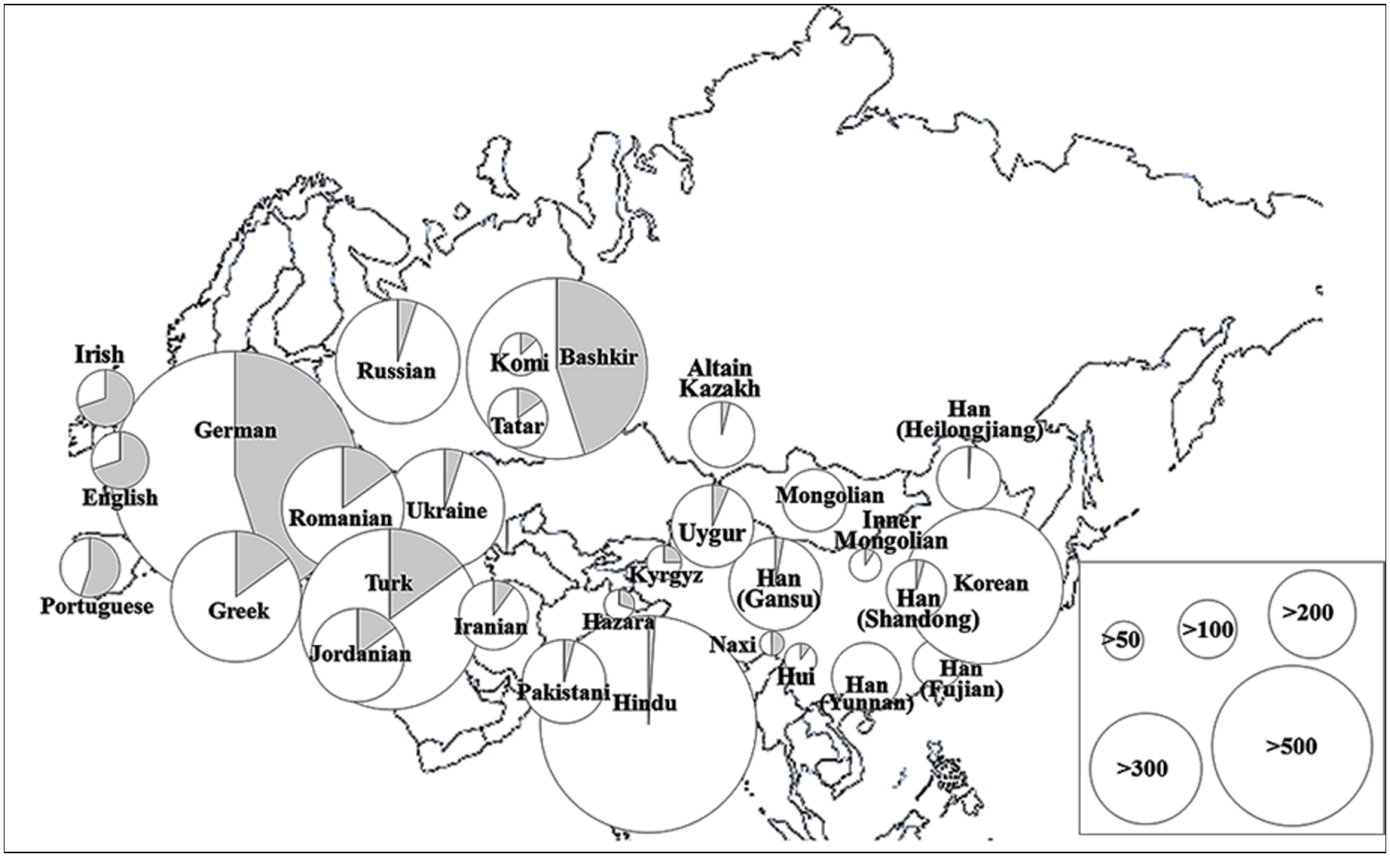
Geographic distribution of modern-day populations with haplogroup R1b-M343. Each circle represents a population sample; the area of the circle is proportional to the sample size. Black sectors denote the relative frequency of R1b-M343-carrying groups identified in the literature.

Eastern Russian Tatars, Bashkirs, and Pakistani Hazara were found to carry R1b-M343 at unusually high frequencies of 12.65%, 46.07%, and 32%, respectively, compared to other regions of Eastern Asia, which rarely have this haplotype (Fig 3) [40, 42, 43, 49–53]. Interestingly, ancestors of those 3 populations were all closely associated with the medieval Mongol Empire. That is, Russian Tatars and Bashkirs are descendants of the Golden Horde (also known as the Ulus of Jochi) that had been controlled by Jochi, the first son of Genghis Khan, and his descendants during the 12^th^–15^th^ centuries. In addition, some of the Hazara tribes are believed to consist of descendants of Mongolian soldiers and their slave women after the 1221 siege of Bamiyan under the leadership of Genghis Khan [54, 55]. Through domination of Hazara, Mongolians strongly influenced the genetic makeup of the Hazara people, especially in Pakistan [49, 54, 56]. Some modern Hazara populations resemble Mongolians in their physical attributes including facial bone structure. Similarly, the high frequency of R1b-M343 in geographic regions associated with the past Mongol khanates including the Golden Horde (from Ural Mountain to Western Siberia, which includes Russia, Ukraine, Belarus, Poland, Azerbaijan, Kazakhstan, and Uzbekistan), Ilkhanate (Iran and neighboring territories including Armenia, Turkey, Georgia, Afghanistan, Syria, and Tajikistan), and Chagatai Khanate (from the Aral sea to the Altai mountain, including Pakistan (Hazara), Uzbekistan, Kazakhstan, Tajikistan, India, and China), strongly suggest a close association between the Y haplotype R1b-M343 and the past Mongol Empire (Fig 3) [42–44, 49–53].

Thus, the appearance of R1b-M343 in Tavan Tolgoi bodies reflects that the genealogical structure of the Genghis Khan’s Golden family consisted largely of a Caucasoid paternal genetic pool, and the distribution of modern-day R1b-M343 carriers at a high frequency in the past Mongol khanates supports that they are direct descendants of Genghis Khan’s Borjigin clan. In contrast, considering that modern-day individuals with specific haplotypes such as C3c-M48 are largely distributed within the past Mongol Empire, Zerjal and colleagues [56] reported that Genghis Khan carried haplogroups C-RPS4Y_711_, and C3c-M48, which are common Mongoloid paternal markers. However, their results did not come directly from the remains of Genghis Khan. Accordingly, nobody can determine whether those haplogroups were indeed carried by Genghis Khan until human remains from Genghis Khan’s grave are excavated to obtain direct proof of the connection of those haplogroups with him.

Collectively, our results provide three possibilities about the high genetic affinity between Tavan Tolgoi bodies and the members of Genghis Khan’s Borjigin clan. First, Tavan Tolgoi bodies would be Golden family members from qudas between the female lineage of Borjigin clan and the male lineage of rulers who dominated Eastern Mongolia, including the Ongud Kingdom. Accordingly, R1b-M343 of Tavan Tolgoi bodies reveals the Y-haplogroup of rulers of Eastern Mongolia in the Mongolian era, not that of Genghis Khan’s Borjigin clan. Second, it is plausible that R1b-M343-carrying Tavan Tolgoi bodies are somehow related to Genghis Khan’s male lineage for a similar reason to C3c-M48 being assumed as the Y-haplogroup of Genghis Khan by Zerjal and colleagues [56]. Thus, Genghis Khan may have carried Y-haplogroup R1b-M343, which is prevalent in West Eurasia, and not haplogroup C3c-M48, which is prevalent in Asia. This is based on Genghis Khan’s physical appearance, which exhibited some features of Caucasoid ethnic groups and the geographical distribution of modern-day R1b-M343 carriers. Third, we cannot entirely exclude the possibility that R1b-M343-carrying modern-day individuals are descendants of Genghis Khan’s generals or relatives who had no genetic relationship with Genghis Khan and his Borjigin clan, but exercised considerable influence throughout the past Mongol khanates including Golden Horde, Ilkhanate, and Chagatai Khanate, as the R1b-M343-carrying modern-day individuals are distributed across the 3 Mongol khanates and are not limited to specific areas, similarly to the Hazara of Pakistan.

### Tavan Tolgoi bodies are members of a family with the relationship of mother-sons or full siblings

As shown in Table 1, mtDNA sequence data for 13 haplogroup-defining coding regions besides HVR1 and HVR2 control regions, in 4 members of the Golden family (MN0104, MN0125, MN0126 and MN0127) matched exactly, with a total of 2,761 base pairs being identical, strongly suggesting that they are immediate family members with the same matrilineal origin. In addition, male bodies, MN0104 and MN0126, were confirmed to be family members based on the results of Y-SNP, Y-STR, and A-STR analyses. MN0127 was also assumed to be their family member, because he shared mtDNA sequences with three other D4-carrying Golden family members; additionally, his burial artifacts and burial location clearly indicate that he was a male with the family relationship with other Golden family members (S1 Table). Additional direct molecular evidence to determine his kinship with other members of Golden family could not be obtained due to failure of PCR amplification for Y-SNP, Y-STR, and A-STR.

Taking these data into consideration, it is clear that 3 (MN0104, MN0125, and MN0126) of the 7 Tavan Tolgoi bodies are immediate family members. In addition, their exact relationship is expected to be 1) a mother (MN0125) and 2 sons (MN0104 and MN0126), 2) a father (1 of the 2 male bodies, MN0104 or MN0126) and 2 children (MN0125 and the other 1 of the 2 male bodies), or 3) 3 siblings. Allelotypes of 30/30 or 30/33.2 at D21S11 marker locus and 19/22 at FGA marker locus in MN0126 could not be obtained from MN0104, assuming that MN0104 is a father of the other 2 bodies (Table 4). Similarly, allelotypes of 29/29 at D21S11 marker locus and 21/27 at FGA marker locus in MN0104 could not come from MN0126, assuming that MN0126 is a father of the other 2 bodies. Due to these conflicting data, we completely ruled out the second possibility. However, the A-STR profiles of 8 marker loci in the 3 Tavan Tolgoi bodies could not rule out the possibility of assumptions 1 and 3. In addition, the results of the Kinship Index Calculation program used by the Korea National Forensic Service for personal identification of Koreans demonstrate that the probability of assumptions 1 and 3 between MN0104 and MN0125 or between MN0125 and MN0126 was nearly 100%. This indicates that the Golden family members, MN0104, MN0125, and MN0126, were either mother-son or had full sibling relationships, assuming that the allelotype of D21S11 was 30/33.2 in MN0126 (S8 Table). The probability of assumptions 1 and 3 between MN0104 and MN0126 was, however, found to be 0% and 64.2%, respectively, indicating no possibility of a parent-child relationship. The probability of assumption 3 decreased to 64.2% because of the small numbers of allelotypes compared, rather than the absence of a relationship between full siblings. In contrast, the probability of kinship among the other 3 Tavan Tolgoi bodies (MN0105, MN0124, and MN0376) and between each of these bodies and the Golden family members (MN0104, MN0125, and MN0126) indicated no family relationship between them. These data strongly suggest that the 3 Golden family members including MN0104, MN0125, and MN0126 are related as either mother-sons or full siblings with nearly the same probability, and have no kinship with the members of the other Golden family (MN0105) and the general public (MN0124 and MN0376).

In any case, it is certain that the grave of the father of the 3 Golden family members, or alternatively a husband of MN0125, was not found near their graves. Accordingly, if assumption 1 is the case, the husband of MN0125 and wives of her possible sons (MN0104 and MN0126) were not buried together with the Golden family members, implying that the Tavan Tolgoi graves were arranged according to the matriarchal, Golden family-dominated burial custom. We cannot exclude the possibility that the husband of MN0125, a guregen, was killed in Genghis Khan’s war and buried somewhere in his death place. This may explain why a Mongol Queen’s husband would not be buried together with his wife and sons. If assumption 3 is true, then it may have been customary for Golden family members to be buried together, far from the graves of their biological parents, particularly from that of their father (i.e., guregen).

### Tavan Tolgoi bodies are Golden family members from marriages between the lineage of Genghis Khan’s Borjigin clan and either the lineage of Ongud clan as guregen or the lineage of Hongirad clan as khatun

Historically, Tavan Tolgoi was dominated by Golden family members, including Alaqai Beki, a daughter of Genghis Khan, Sorghaghtani, the wife of Tolui, Genghis Khan’s youngest son, and their descendants or successors [2]. Taking into account all the data in this study, several scenarios could explain the identity of the Golden family members excavated in Tavan Tolgoi. First, MN0125 who is regarded as a Mongol Queen can be assumed to be Alaqai Beki, who ruled the Ongud Kingdom instead of her husband, the guregen. This scenario, however, can be easily refuted because Alaqai Beki had only one son and died approximately in her 50s; MN0125 was buried together with 3 sons or 3 full siblings and died in her 20s (S1 Table). In addition, if MN0125 is Alaqai Beki, a sister of other three males (MN0104, MN0126 and MN0127), her male siblings would be the Great Khans, including Ogodei, the 2^nd^ Great Khan of the Mongol Empire. Of course, this assumption is not also acceptable. In the second scenario, MN0125 can be presumed to be Sorghaghtani. It is illogical, however, that the 3 male graves at Tavan Tolgoi are those of the Great Khans, her sons, Mongke and Kublai, the 4^th^ and 5^th^ Great Khans of the Mongol Empire, respectively. In addition, the presumed age at death of MN0125 is inconsistent with that of Sorghaghtani, who died in her late 60s [57]. Moreover, Sorghoghtani was a Christian, and was buried in a church located in the Gansu province, Northwestern China, far from Tavan Tolgoi [57]. Accordingly, the assumption that MN0125 is Sorghoghtani is excluded. Third, the Tavan Tolgoi bodies could be direct descendants of Alaqai or Sorghaghtani. This possibility, however, is also untenable because their descendants were died young or could not be Great Khans as mentioned above. Fourth, Tavan Tolgoi bodies may be successors of Alaqai. Alaqai invented a new marriage alliance system, quda, between women, probably her close relatives, of Genghis Khan’s Borjigin clan and men of the ruling lineages of the Ongud, to solidify her political power and to extend her rule over the Ongud Kingdom [2, 3]. Accordingly, it is possible that the Tavan Tolgoi graves belong to the ruling lineage newly formed by qudas, as successors of Alaqai Beki, who had no sons. Fifth, the Tavan Tolgoi graves may belong to the Hongirad clan. The homeland of the Hongirad clan was located near Hulun Lake, Northeastern Mongolia, which is not far from Tavan Tolgoi [11]. During the Yuan dynasty, some Hongirads moved to the territory of modern Uzbekistan and Kazakhstan; today their descendants are widely distributed in the modern Kazakh, Western Mongolian, Uyghur, and Uzbek people. Women of the Hongirad clan married many Great Khans of the Borjigin clan, including Genghis Khan and his descendants, as well as his ancestors [8]. The qudas between the male lineage of the Borjigin clan and the female lineage of the Hongirad clan continued in the Yuan Dynasty and the Golden Horde Dynasty, giving the Hongirad tribe enormous power in the Mongol Empire as a clan of Mongol khatuns including Genghis Khan’s grandmother, mother, and chief wife, Borte Khatun [2, 3, 8]. Accordingly, it is possible that some Golden family members from quads between the male lineage of Genghis Khan’s Borjigin clan and the female lineage of khatuns of the Hongirad clan were buried in a sacred burial site, such as Tavan Tolgoi.

MN0105 is presumed to be the most important figure among the Tavan Tolgoi bodies. The Mongolian archaeologist, Dr. Navaan D., who first excavated the Tavan Tolgoi graves suggested the possibility that MN0105 is a mother of MN0104, since MN0104 was buried near the foot of MN0105 and the presumed age of death of MN0104 was approximately his late 20s whereas that of MN0105 was around the age of 45–55 years [9, 10]. At the time of excavation, the archaeological importance of MN0105 was overlooked in favor of MN0125 because MN0105 was buried with only a modest amount of artifacts, including 2 golden rings and ornaments for boqta, whereas MN0125 was unearthed with numerous expensive and interesting artifacts. Nonetheless, MN0105’s burial artifacts, such as golden rings engraved with the falcon image, clearly indicate that she is closely related to Genghis Khan’s Borjigin clan. If we assume that MN0105 is Alaqai Beki or another female ruler from Genghis Khan’s Borjigin clan, then she should be related politically, not biologically, to the other Golden family members from Tavan Tolgoi as their stepmother, foster mother, or guardian. In other words, it is likely that they would be from a quda between the female lineage of Genghis Khan’s Borjigin clan and the male lineage of the Ongud clan, arranged by Alaqai Beki who had no direct descendants, or by a female ruler from Genghis Khan’s Borjigin clan.

Considering the historical, archaeological, physical anthropological, and molecular archaeological evidence obtained, it seems most likely that the Tavan Tolgoi bodies are members of Genghis Khan’s Golden family, including the lineage of bekis, Genghis Khan’s female lineage, and their female successors who controlled Eastern Mongolia in the early Mongolian era instead of guregens of the Ongud clan, or the lineage of khans, Genghis Khan’s male lineage, who married females of the Hongirad clan, including Genghis Khan’s grandmother, mother, chief wife, and some daughters-in-law.

### The modern-day descendants of Tavan Tolgoi bodies have disappeared from the Mongolian plateau

We found that 27.8% (15/54) modern-day Mongolians carry the mtDNA haplogroup D4 at about (S9 and S10 Tables). Keyser-Tracqui and colleagues [58] and Kim and colleagues [18] also reported that D4 was found in about 36.96% among Northern Mongolian populations in the Xiongnu age, and in 2 of 3 Xiongnu bodies in the North Eastern Mongolia. This implies that the mtDNA haplogroup D4 is one of the most prevalent haplogroups across the Mongolian plateau from at least the Xiongnu era to the present. In comparison, our unpublished data demonstrated that the Y-haplogroups R1b-M343 and R1a1a-M17 are distributed at 0.0% (0/101) and 0.99% (1/101) in modern-day Mongolians across the Mongolian plateau, respectively (S10 Fig) [31, 32]. Zhong and Colleagues [50] also reported that the modern-day Mongolians who inhabit in the Inner and Outer Mongolia carry the R1b-M343 haplogroup at 8.3% (1/12) (only in Heilongjiang; the province located in the North Eastern part of China) and 0.0%, respectively. Meanwhile, Zhong and colleagues [50] and Katoh and colleagues [59] demonstrated that the R1a1a-M17 was found at 9.1% (2/22), 3.5% (3/85), 6.7% (4/60) and 13.3% (8/60) in modern-day Inner Mongolians, Khalkh, Uriankhai, and Zakhchin Mongolian tribes, respectively. Thus, R1b-M343 is scarcely found in the Mongolian plateau, whereas R1a1a-M17 is widely distributed, although at a relatively low frequency, having a maximum of 13.3% in the Zakhchin tribe [59]. These results demonstrate that modern-day individuals carrying R1b-M343 are hard to find on the Mongolian Plateau, meaning that descendants of R1b-M343-carrying members of the Golden family disappeared from the Mongolian Plateau for unknown reasons.

Modern-day individuals with the same Y-STR profiles as members of the Golden family have been carefully screened in many studies and in YHRD from modern-day individuals, totaling approximately 154,329 individuals (searched on August 25, 2015). Modern-day individuals matching the Golden family members in Y-STR profiles from Yfiler and PowerPlex Y of YHRD and the literature are mainly distributed in Kalmykia, Russia, Uzbekistan, Tajikistan and China (Fig 3 and S6 Table).

Coincidentally, the geographical distribution of modern-day individuals matching the Y-haplogroup and haplotype of the Tavan Tolgoi bodies in the regions corresponding to the past Mongol khanates, including the Golden Horde Dynasty and Chagatai Khanate, implies that the modern-day individuals are direct descendants of the Golden family members. The ancestors of Kalmykia are the Oirats, the westernmost tribe of the Mongols. The Oirats also had strong ties with Chagatai Khanate and the Golden Horde, through marriage alliances between Mongol khans and Oirat khatuns, just like the Hongirads [2, 60]. These distributions imply the movement of descendants of the Golden family from Eastern Mongolia to West Eurasia, including Kalmykia, and a possible genealogical connection between Golden family members and the Oirats. By the 9^th^ century, the Shato Turks of the Western Göktürks Khaganate, as ancestors of the Ongud, moved to modern-day Inner Mongolia and eventually were dominated by the Mongol Oirats, later known as Kalmyks, suggesting an anthropological connection of the Ongud with Kalmyks [33, 45, 46, 61, 62]. Taken together, Golden family members from Tavan Tolgoi may have been direct ancestors of R1b-M343-carrying modern-day individuals who live in the territories of the past Mongol khanates.

Why both R1b-M343 carriers and modern-day individuals with the same Y-STR profile as that of the Golden family members are rarely found in the Mongolian plateau could be explained by the following 2 hypotheses, which are not mutually exclusive. One is large-scale redeployment of descendants of our Golden family members from the Mongolian Plateau to Eastern Europe (Kalmykia and Russia) or Central Asia (Uzbekistan and Tajikistan). Many of the Onguds returned to the ancestral homeland near Central Asia from Eastern Mongolia; this turn of events resulted in a significant decrease in the number of their descendants, including R1b-M343 carriers, in Eastern Mongolia. The other possibility is internecine massacre among direct male descendants of Genghis Khan’s Borjigin clan and their wives (i.e., among the Golden family). As soon as Genghis Khan died, kingdoms of bekis, including the Ongud, were attacked and eventually toppled by the daughters-in-law and grandsons of Genghis Khan [2, 3]. Most bekis lost power and were killed in a horrendous manner by opponent factions including the Great Khans, such as Ogodei and Mongke [2]. Under such political conditions, most Golden family members, including the lineages of the former rulers of the Ongud or the Hongirad, who were opposed to the faction in power, were likely exterminated [2, 3]. Meanwhile, Golden family members who lived in the Golden Horde, Ilkhanate and Chagatai Khanate where are far apart from the central area of the Mongol Empire were relatively safe from such horrendous massacre.

Thus, the large-scale movement and slaughter that occurred in the Mongolian plateau could explain at least in part why direct descendants of the Golden family are hard to find in modern-day Mongolia. However, further studies are needed to firmly conclude when and why R1b-M343 carriers, which are distributed mostly in Europe and Central Asia, appeared and then subsequently disappeared from the Mongolian Plateau region.

## Materials and Methods

### Subjects

The basic characteristics of the 7 graves excavated from the center hill of Tavan Tolgoi are shown in S1 Table. In all 7 graves, human skeletons with skulls were excavated. Skulls, femurs, and/or pelvic bones were used for physical anthropological analysis, and femurs or humeri for molecular archaeological analysis. All 7 Tavan Tolgoi human remains and 122 contemporary buccal materials were provided by D. Tumen, one of authors, with permission (No. 10-23-2213) from the Mongolian government for authority to take human materials out of the country and to carry out molecular archaeological study in 2006. When molecular archaeological approaches were carried out using human materials, the Mongolian and Korean governments had no specified regulation system (such as IRB and Ethics Committee) required for scientific works using human materials, and therefore no additional permits were required for this study.

A total of 122 contemporary individuals who have no family relationships with each other and who are from varied ethnic groups were subjected to buccal swab analysis to determine their matrilineal and/or patrilineal origins. All 7 skeletons used in this study and deposited in the National University of Mongolia are publicly available with restricted use, as ancient human skeletons excavated in Mongolia are regarded as cultural assets and the amount available for scientific research is limited. Accordingly, researchers who are interested in using Tavan Tolgoi samples in countries outside of Mongolia must obtain the approval of the Mongolian government for foreign transfer of these samples and the permission of the authors K-H Lee and D. Tumen.

### Physical anthropological analysis

The skeletons of Tavan Tolgoi bodies were evaluated for physical anthropological parameters, including the morphometric, osteometric, and/or odontometric characteristics of the skull, pelvic bone, and femur (S2 Table). Cranial measurement of the skulls was performed as described by Martin [63]. Estimation of the height and weight of the bodies from the graves was carried out using femurs according to the equations described by Pearson [64]. The methods proposed by Nakahashi and Nagai [16] and Brothwell [17] were used to estimate anatomical sex and age at death. Relative dating of burial artifacts from Tavan Tolgoi graves was performed by conventional methods commonly used in the field of archaeology.

### Extraction and purification of aDNA

Extraction and purification of aDNAs from human and horse skeletons were carried out using our previously published method, which ensures both sufficient quality and quantity of aDNA for PCR amplification [18, 65]. Briefly, bone fragments preimmersed in bleach and irradiated with UV were crushed to make bone powders using the Mixer Mill MM301 (Retsch GmbH and Co., Haan, Germany). The bone powders were incubated with extraction buffer QG (Qiagen Co., Valencia, CA, USA). After incubating the supernatant with silica particles, the silica extracts were purified using ion exchange columns with QBT, QF, and QC buffers (Qiagen Co.). Elutes were concentrated up to 150 μL using the QIAquick purification kit (Qiagen Co.).

Purified aDNAs were routinely quantified by comparing individual curves to a standard curve, and ion exchange columns were used to eliminate PCR inhibitors from aDNA extracts that often lead to PCR failure [18, 65]. Despite pretreatments such as quantification and ultra-purification of aDNAs, if the resulting amount of purified aDNAs was extremely low and/or aDNA extracts purified or products of the first round PCR were assumed to contain PCR inhibitors, we re-purified aDNAs from bone powders or re-amplified the aDNAs using primer sets designed for nested PCR amplification.

### Contamination precautions

To prevent potential contamination, aDNAs from bone samples were collected and amplified by PCR with extensive precautions in two separate, clean rooms for pre- and post-PCR experiments, which were specifically dedicated to aDNA only. The clean rooms were equipped with positive pressure and air filtration system, and all people handling the materials or working in the laboratory wore protective clothing, including UV-irradiated lab gowns, face and mouth masks, and latex gloves. All materials, including tubes and pipettes equipped with aerosol resistant tips, were autoclaved or sterilized, and work places were cleaned with bleach and UV irradiated at 254 nm for at least 1 hour. Repeated extractions and PCR-amplifications of aDNA were carried out in DNA-free reagents. Mock extractions without samples and PCR blanks were used to carefully monitor contaminations of aDNA from researchers and other organisms throughout the experiment.

### UDG treatment

A 10-μL aliquot of each aDNA extract was treated with 1 U of uracil-DNA glycosylase (UDG; Roche Diagnostics Co., Indianapolis, IN, USA) before PCR amplification, while another aliquot was treated with 1 U of UDG that was first heat-inactivated at 95°C for 10 min. The aDNA treated with active or inactive UDG was cloned into the TA cloning vector provided in the pGEM-T Easy Vector System I TA cloning kit (Promega, Madison, WI, USA) according to the manufacturer’s instructions. DNA sequences were then analyzed in at least 4 and 5 clones for each sample from active UDG and inactive UDG samples, respectively. After treatment with active or inactive UDG, sequence data were compared to determine whether the cloned sequences retained sporadic base substitutions (C→T/G→A).

### mtDNA analysis

To define haplogroup affiliations, the D-loop HVR1 (positions 15,977–16,399), HVR2 (positions 29–381), and 10 coding regions of mtDNA were amplified by using primer sets presented in S5 Table and sequenced [18, 23, 66]. The primer set used to amplify the coding region containing SNPs for mtDNA haplogroup M was also used to determine the mtDNA haplogroups N and Z; an amplicon produced by the primer set for haplogroup M contained SNP sites for haplogroups N and Z, as well as for M. In addition, the coding region containing SNPs for the mtDNA haplogroups CZ and G was simultaneously amplified by a primer set for CZ. Accordingly, a total of 13 haplogroup-defining SNPs were typed through DNA sequence analysis of mitochondrial coding regions amplified by the 10 primer sets.

HVR1 and -2 PCRs were performed in a 20 μL reaction mixture containing 2 μL template DNA, 2 μL 10× PCR buffer (Applied Biosystems Inc., Foster City, CA, USA), 0.2 mM dNTP mix, 2 mM MgCl_2_, 1 μM each primer, 1 mg/mL BSA (New England Biolabs Ltd., Hitchin, UK), and 1.5 U/μL AmpliTaq Gold polymerase (Applied Biosystems Inc.). A T3 thermocycler PCR system was used with the following conditions: 95°C for 10 min; 40 cycles of 95°C for 30 s, 56°C–60°C for 1 min, 72°C for 1 min; and a final extension at 72°C for 7 min. Amplification of coding regions was performed in a 25 μL reaction mixture consisting of 1.5 μL template DNA, 5 μL 2× PCR buffer, 0.2 mM dNTP, 0.1 mg/mL BSA, 0.25 pM of each primer, and 4 U/μL Super-Therm Gold Taq polymerase (Bertec Enterprise Co. Ltd., Taipei, Taiwan). A T3 thermocycler PCR system was used with the following conditions: 95°C for 5 min, 42 cycles of 95°C for 1 min, 56°C–60°C for 1 min, 72°C for 30 s; and a final extension at 72°C for 7 min. PCR products with low quality and quantity were reamplified in a nested PCR reaction mixture under the same PCR conditions mentioned above but with the first PCR products diluted 1:50 for an additional 25 cycles. PCR products were purified using the RBC PCR purification kit (RBC Bioscience Co., Taipei, Taiwan) and bidirectionally sequenced at the Macrogen Service Center (Macrogen Inc., Seoul, Korea).

### Y-SNP analysis

Male Tavan Tolgoi bodies were tested for haplogroup-defining SNPs using a set of 10 biallelic markers, with reference to the hierarchical order based on the global Y-haplogroup distribution [18, 31, 67–69]. In the first step, biallelic markers defining the macro-haplogroups O, C, D, and N, which are known to be widely distributed in East and Northeast Asia, were applied to determine the Y-hierarchical orders of the Tavan Tolgoi bodies. Next, other haplogroups or their subclades, such as J, Q, R, R1, R1b and R1a1a, were determined.

All the primers used in this study were redesigned to ensure amplicons were as small as 127–231 base pairs to facilitate PCR amplification of highly fragmented aDNAs (S5 Table) [18]. The target biallelic markers were amplified in monoplex or multiplex reactions. A group of biallelic markers consisting of C-RPS4Y_711_, D-M174, and N-M231, and the other group consisting of J-M304, Q-M242, and R-M207 were used in multiplex I and multiplex II, respectively [18]. Each of the remaining biallelic markers for O-M175, R1-M173, R1b-M343 and R1a1a-M17 was amplified separately in monoplex reactions [18]. PCR was performed in a 10 μL final volume composed of 1 x PCR Gold Buffer (Applied Biosystems Inc.), 2 mM MgCl_2_, 0.2 mM dNTP, 1 mg/ml BSA (New England Biolabs Ltd.), 0.4 U/μL AmpliTaq Gold DNA polymerase (Applied Biosystems Inc.), 0.75 μM primer, and 4 μL aDNA template. Thermal cycling consisted of a first denaturation step at 95°C for 11 min followed by 45 cycles of 94°C for 30 s, 60°C for 1 min and 72°C for 1 min with a final extension at 72°C for 7 min. Each marker was amplified again in a nested PCR reaction under the same cycling condition as mentioned above but with the first PCR products diluted 1:50 for 26 cycles instead of 45 cycles. Amplicons were sequenced in both directions.

### Y-STR analysis

Male Tavan Tolgoi bodies were tested for 16 Y-chromosomal STR loci [DYS19, DYS385, DYS389I, DYS389II, DYS390, DYS391, DYS392, DYS393, DYS437, DYS438, DYS439, DYS448, DYS456, DYS458, DYS635 (Y GATA C4) and Y GATA H4] using the AmpFlSTR^®^ Y-filer^™^ PCR Amplification Kit (Applied Biosystems Inc.). All procedures used for Y-STR analysis were carried out according to the manufacturer’s protocol with minor modifications in PCR conditions, including increased concentrations of templates and 35 PCR cycles rather than 30 cycles. STR products were analyzed on an ABI Prism 3100 Genetic Analyzer using GeneMapper software (Applied Biosystems Inc.).

### A-STR analysis

A-STRs were amplified using the AmpFlSTR^®^ MiniFiler^™^ PCR Amplification Kit (Applied Biosystems Inc.). Eight STRs (D2S1338, D7S820, D13S317, D16S359, D18S51, D21S11, CSF1PO, and FGA) and the sex-determining marker amelogenin were simultaneously amplified. PCR amplification of A-STRs was performed according to the manufacturer’s protocol with minor modifications in PCR conditions, including increased concentration of templates and 35 PCR cycles rather than 30 cycles. Capillary electrophoresis was conducted on an ABI Prism 3100 Genetic Analyzer (Applied Biosystems Inc.) for allelotyping of the Tavan Tolgoi bodies. PCRs were performed in a maximum of 9 repeats (6 repeats in the first laboratory and 3 in the second laboratory) for each sample. When identical data were obtained from two independent laboratories, the deduced data were used for further analysis. Data analysis was performed using GeneMapper software (Applied Biosystems, Inc.) and the Kinship Index Calculation program, which was developed by the Korea National Forensic Service and has been extensively used for personal identification. The Kinship Index Calculation program was developed based on the Identity-By State and likelihood ratio assessment [70, 71].

### Determination of haplotypes and haplogroups of aDNA and statistical analysis

mtDNA sequence data were analyzed using SeqMan II software (DNASTAR, Madison, WI, USA) and compared to rCRS [21]. Possible mutations were verified in the GenBank database and Genographic Project database. Haplogroups of aDNAs were first determined by analyzing mtDNA sequences of HVR1 and HVR2 using the well-established web-based program, mtDNAmanager [24]. To identify particular haplogroups not clearly determined using the control regions (HVR1 and HVR2), 13 haplogroup-defining SNPs within 10 mitochondrial coding regions were additionally typed. As shown in S5 Table, many primer sets for mtDNA haplogroups and subclades were selected from our previous report [18].

The geographical and ethnic distribution of modern populations with the same mtDNA haplotypes as the Tavan Tolgoi bodies was elucidated via comparison of HVR1 and HVR2 sequences of the Tavan Tolgoi bodies with those of modern individuals who were previously characterized based on their HVR1 and HVR2 sequences [18]. The most closely related mtDNA haplotypes among the Tavan Tolgoi bodies and 8,478 modern individuals from the GenBank database, including our modern-day Mongolians, were analyzed by constructing an NJ tree with concatenated HVR1 (positions 16,024–16,380) and HVR2 (positions 47–360) sequences using ClustalW 2.0.11 [18, 72, 73]. The NJ tree was drawn using MEGA version 6.0.6 [39, 74]. Modern mtDNA sequences proximal to the aDNA sequences were further analyzed by reconstructing a bootstrapped NJ tree calculated from 1,000 resamplings of the alignment data [18].

Y-haplogroups were determined based on a recently revised Y-haplogroup tree [31, 68]. The Y-SNP results were used to determine the patrilineal origins of male bodies from Tavan Tolgoi and the geographic distribution of modern-day individuals with the same Y-haplogroup as those of Tavan Tolgoi bodies. In addition, the Y-STR profiles served 2 purposes: 1) to assess kinship among the Tavan Tolgoi bodies and 2) to compare their haplotypes with those of modern human populations found in the YHRD and the literature to identify their direct descendants. Similarity among male members of Tavan Tolgoi in the Y-STR profiles of 16 marker loci was examined to determine possible kinship among samples. To screen for Y-STR profiles that were identical between the Tavan Tolgoi bodies and modern-day individuals from YHRD (a maximum of 154,329 individuals; searched on August 25, 2015), the allelotypes of 11 and 16 Y-STR marker loci were defined using PowerPlex Y and Yfiler of YHRD, respectively. Moreover, the Y-STR profiles of Tavan Tolgoi bodies were screened to identify modern-day individuals with the same Y-STR profiles based on data from previous studies of Y-STR profiles associated with modern Eurasian populations. Both the results from YHRD and the literature were applied to determine the geographical distribution of direct modern-day descendants of Tavan Tolgoi bodies. To analyze the kinship among Golden family members, pairwise comparison of their A-STR profiles was also conducted using the Kinship Index Calculation program.

## Supporting Information

S1 Fig PCR amplification of ancient horse mtDNA. A: Agarose gel electrophoretic analysis of mtDNA amplified from aDNA extracted from molars of a horse excavated together with MN0105. B: DNA sequencing data from PCR products (368 bp) amplified from molars of a horse using primers for horse mtDNA HVR1. PCR products were successfully obtained using primers for horse HVR1 as shown in lanes 1–4; no amplicons were obtained using primers for human HVR1 (F15971/R16410) as shown in lanes 5–8, confirming the specificity of our PCR experiments. Underlining indicates nucleotide sequences of the primers used. M: 100-bp DNA ladder, DW: distilled water.
(TIF)

S2 Fig DNA sequence data for identification of a Y-haplogroup (MN0104). ^a^Bold characters indicate the nucleotide positions of the haplogroup-defining SNPs. ^b^Boxes of dotted and solid lines indicate no substitution and specific mutations, respectively, in the haplogroup-defining SNPs. Hp: haplogroup, and NC: not changed.
(TIF)

S3 Fig DNA sequence data for identification of a Y-haplogroup (MN0126). ^a^Bold characters indicate the nucleotide positions of the haplogroup-defining SNPs. ^b^Boxes of dotted and solid lines indicate no substitution and specific mutations, respectively, in the haplogroup-defining SNPs. Hp: haplogroup, AF: failure of PCR amplification, and NC: not changed.
(TIF)

S4 Fig DNA sequence data for identification of a Y-haplogroup (MN0376). ^a^Bold characters indicate the nucleotide positions of the haplogroup-defining SNPs. ^b^Boxes of dotted and solid lines indicate no substitution and specific mutations, respectively, in the haplogroup-defining SNPs. Hp: haplogroup, NC: not changed, and del: deletion of the base indicated.
(TIF)

S5 Fig Y-STR electropherograms of male members of Golden family. A: MN0104. B: MN0376.
(TIF)

S6 Fig Geographical and ethnic distribution of modern-day individuals with the same mtDNA haplotype as the Tavan Tolgoi bodies (MN0104, MN0125, MN0126, and MN0127) in the constructed neighbor-joining tree. ^a^mtDNA haplogroups were determined by means of mtDNAmanager, a Web-based tool for the management and quality analysis of mtDNA sequences of control regions.
(TIF)

S7 Fig Geographical and ethnic distribution of modern-day individuals with the same mtDNA haplotype as that of a Tavan Tolgoi specimen (MN0105) in the constructed neighbor-joining tree. ^a^mtDNA haplogroups were determined by means of mtDNAmanager, a Web-based tool for the management and quality analysis of mtDNA sequences of control regions. Minus (-) indicates that no information is available about the population origin.
(TIF)

S8 Fig Geographical and ethnic distribution of modern-day individuals with the same mtDNA haplotype as that of a Tavan Tolgoi specimen (MN0124) in the constructed neighbor-joining tree. ^a^mtDNA haplogroups were determined by means of mtDNAmanager, a Web-based tool for the management and quality analysis of mtDNA sequences of control regions.
(TIF)

S9 Fig Geographical and ethnic distribution of modern-day individuals with the same mtDNA haplotypes as those of a Tavan Tolgoi specimen (MN0376) in the constructed neighbor-joining tree. ^a^mtDNA haplogroups were determined by means of mtDNAmanager, a Web-based tool for the management and quality analysis of mtDNA sequences of control regions.
(TIF)

S10 Fig Hierarchical distribution of Y-haplogroups in modern-day Mongolians. Y-haplogroups and subclades were determined using the Y-haplogroup tree of International Society of Genetic Genealogy.
(TIF)

S1 Table Various characteristics of the Tavan Tolgoi graves. ^a^Data published by Youn and colleagues [14]. According to their ^14^C radiocarbon dating results, the fragments of the wooden coffin of MN0127 were dated 230–540 AD. Because cinnamon usually takes 60 years to grow to a diameter of 60 cm, and therefore several hundred years to reach dimensions of 2–3 m in diameter, those authors supposed that the reason for the earlier dating of MN0127 compared with the other Golden family members could be explained by such a slow growth rate of the cinnamon. ^b^Results of physical anthropological analyses. ^c^The golden rings were engraved with the falcon image that symbolizes Genghis Khan and his Borjigin clan. ^d^MN0124 was disinterred without any noticeable artifacts except small golden earrings, according to the Mongol tradition that antenuptial children who do not own property were to be buried without any burial artifacts. ^e^Pearl and golden ornaments of boqta showing the same design and shape as those of Mongol khatuns. ^f^In the golden container, there were some brownish red powders that were presumed to be some kind of medicine or incense. ^g^A bronze mirror assumed to be related to Buddhism with Sanskrit writing. ^h^The golden vajra (thunderbolt) that was in the hand of MN0125 and is known to be related to Buddhism. ^i^The golden saddle was sheathed with dragon-shaped decorations; to date, such exquisite discoveries have never been found in Mongolia. ^j^The golden earring that Mongolian male aristocrats used to wear in the left ear. ^k^The wooden coffin was made from the cinnamon plant (*Cinnamomum sp*.) that would have been transported from the Southern part of Asia. ^l^The single golden earring similar to that of MN0126 in shape and design. ND; not done.
(DOCX)

S2 Table Osteometric parameters of skulls of the Tavan Tolgoi bodies. ^a^Estimation of cranial metric traits was conducted according to the method proposed by Martin [63]. Minus (-) indicates inability to estimate cranial metric traits due to partial breakage of a skull. Data from MN0125 and MN0127 are not available because their skulls were badly broken and did not allow for estimation of cranial metric traits.
(DOCX)

S3 Table Among the Tavan Tolgoi bodies (MN0104, MN0125, and MN0126), comparison of HVR1 nucleotide sequences between direct PCR products and clones obtained after treatment with either active or inactive uracil DNA glycosylase (UDG). ^a^Nucleotide sequences of amplicons were obtained using PCR-directed sequencing of mtDNA HVR1. Minus (-) and blank indicate failure of PCR amplification and failure to clone PCR products, respectively. PCR-directed sequencing data were identical to the sequencing data from all active and inactive UDG–treated clones in their consensus sequences. Con: consensus sequence.
(DOCX)

S4 Table Among the Tavan Tolgoi bodies (MN0105, MN0124, and MN0376), comparison of HVR1 nucleotide sequences between direct PCR products and clones obtained after treatment with active and inactive uracil DNA-glycosylase (UDG). ^a^Nucleotide sequences of amplicons were obtained by PCR-directed sequencing of mtDNA HVR1. Minus (-) and blank indicate failure of PCR amplification and failure to clone PCR products, respectively. All PCR-directed sequencing data were identical to the sequencing data from active UDG-treated clones in their consensus sequences, except for the C→T substitution (the box in the table) in the 5 clones derived from inactive UDG-treated PCR products of MN0376. A solid box indicates the substituted base; base T was converted to base C after incubation with active UDG, meaning that aDNA from MN0376 had postmortem damage (C→T) and the damaged base was restored to the authentic base C by UDG. Con: consensus sequence.
(DOCX)

S5 Table Primers for identification of haplogroups of mtDNA and the Y-chromosome. ^a^Nomenclature of mtDNA haplogroups is based on the updated tree of mtDNA haplogroup. Numbers indicate nucleotide positions based on the revised Cambridge Reference Sequence (rCRS). ^b^Primer set used for amplification of HVR1 in clones obtained after treatment with UDG. ^c^Primer set used for amplification of haplogroup (M, N and Z)-defining SNP sites. ^d^Primer set used for amplification of haplofroup (CZ and G)-defining SNP sites. ^e^Nomenclature of Y-haplogroups is based on the revised haplogroup tree.
(DOCX)

S6 Table Modern-day individuals with the Y-STR profile matching that of the Tavan Tolgoi body (MN0104). ^a^Ge. Hp: Genotyped Haplogroup, Haplogroup previously determined by Y-SNP analysis in the literature indicated. ^b^YHRD: Y-Chromosome STR Haplotype Reference Database. ^c^Pre. Hp: Predicted Haplogroup, Haplogroup predicted using the Yfiler and PowerPlex Y of YHRD, based on the Y-STR profiles. Hp: haplogroup, NM: No match, ND: Not done, and NDT: Not determined.
(DOCX)

S7 Table Modern-day individuals with the Y-STR profile matching that of the Tavan Tolgoi body (MN0376). ^a^Ge. Hp: Genotyped Haplogroup, Haplogroup previously determined by Y-SNP analysis in the literature indicated. ^b^YHRD: Y-Chromosome STR Haplotype Reference Database. ^c^Pre. Hp: Predicted Haplogroup, Haplogroup predicted using the Yfiler and PowerPlex Y of YHRD, based on the Y-STR profiles. Hp: haplogroup, NM: No match, ND: Not done, and NDT: Not determined.
(DOCX)

S8 Table Kinship analysis among Tavan Tolgoi bodies using the Kinship Index Calculation program. ^a^The probabilities expressed as percentages were rounded to two decimal places. ^b^Allelotype of D21S11 in MN0126. UR: unrelated.
(DOCX)

S9 Table Distribution of mtDNA haplotypes and haplogroups of modern-day Mongolians. mtDNA haplogroups were determined by means of mtDNAmanager, a Web-based tool for the management and quality analysis of mtDNA sequences of control regions. Hp: haplogroup.
(DOCX)

S10 Table Ethnic distribution of modern-day Mongolians selected to determine the haplogroups of mtDNA and Y chromosome. ^a^Ethnic distribution of individuals subjected to buccal swab analysis. ^b^Percentage of ethnic groups in all Mongolians was presented based on Mongolia population census data (2010).
(DOCX)
