# Supplementary figures and images for: Molecular Genealogy of a Mongol Queen’s Family and Her Possible Kinship with Genghis Khan
Source: PLoS One. 2016 Sep 14;11(9):e0161622. doi: 10.1371/journal.pone.0161622 (PMC5023095; doi:10.1371/journal.pone.0161622)

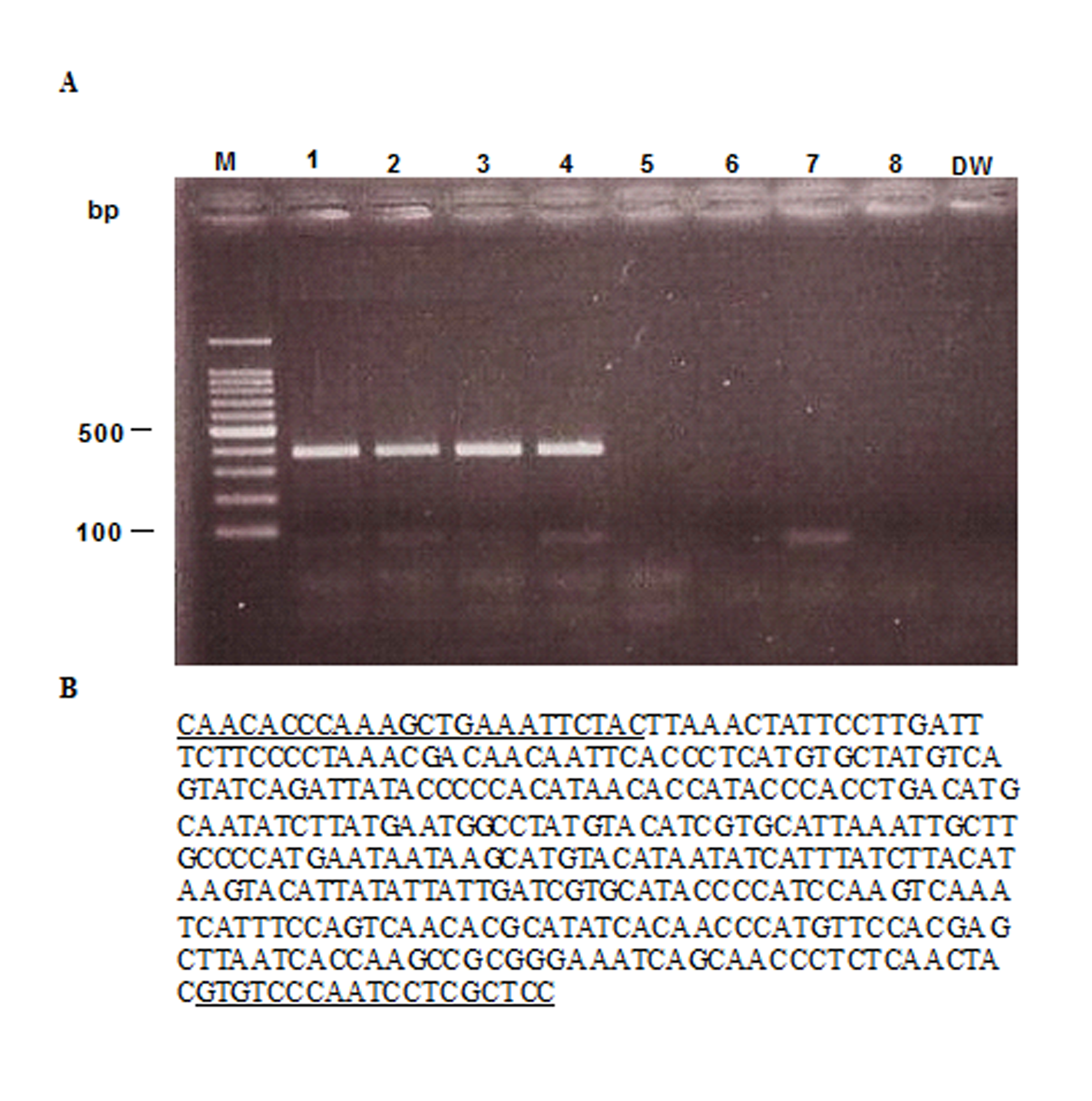

Supplement: S1 Fig — A: Agarose gel electrophoretic analysis of mtDNA amplified from aDNA extracted from molars of a horse excavated together with MN0105. B: DNA sequencing data from PCR products (368 bp) amplified from molars of a horse using primers for horse mtDNA HVR1. PCR products were successfully obtained using primers for horse HVR1 as shown in lanes 1–4; no amplicons were obtained using primers for human HVR1 (F15971/R16410) as shown in lanes 5–8, confirming the specificity of our PCR experiments. Underlining indicates nucleotide sequences of the primers used. M: 100-bp DNA ladder, DW: distilled water. (TIF) [file pone.0161622.s001.tif]

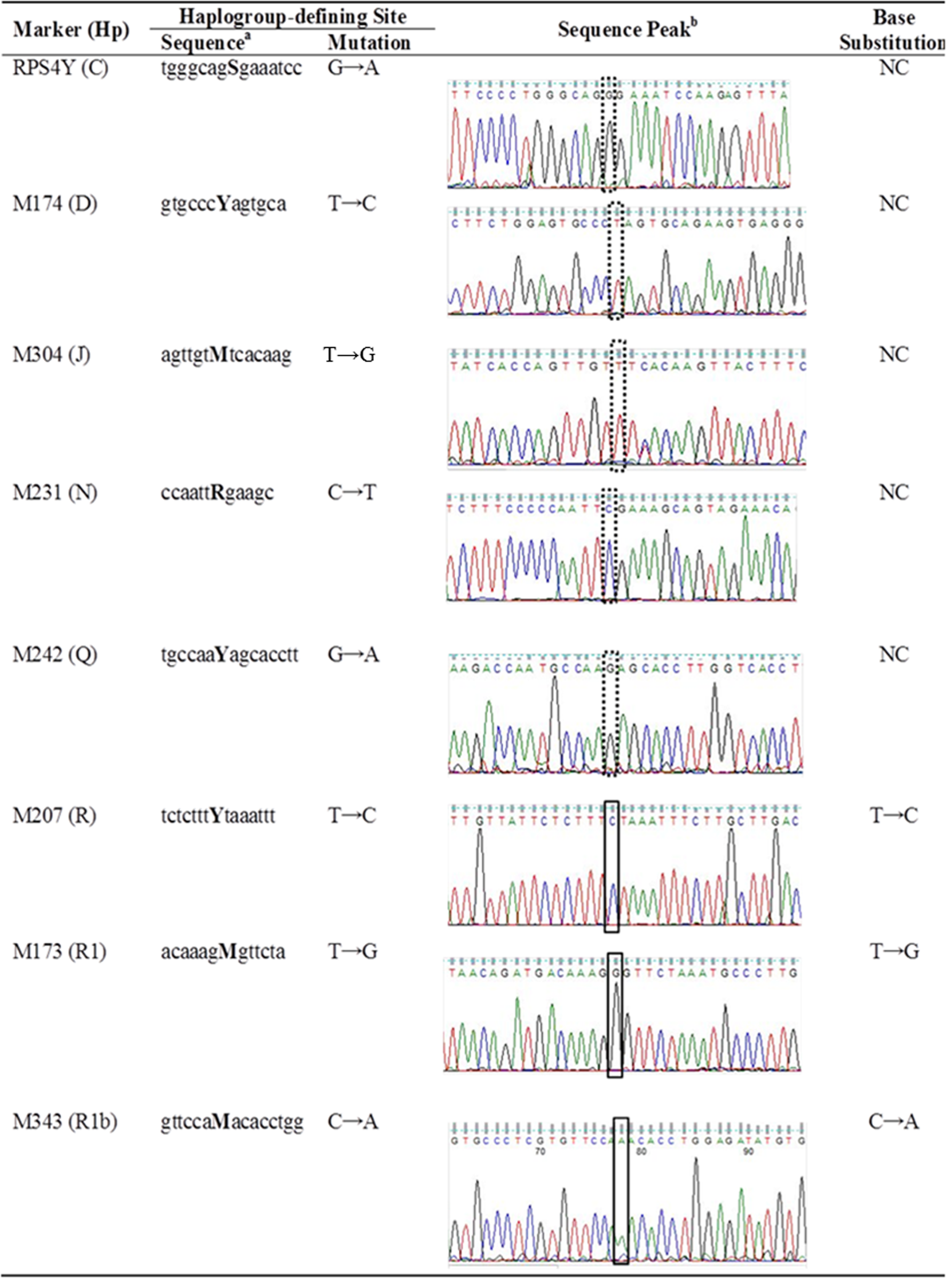

Supplement: S2 Fig — aBold characters indicate the nucleotide positions of the haplogroup-defining SNPs. bBoxes of dotted and solid lines indicate no substitution and specific mutations, respectively, in the haplogroup-defining SNPs. Hp: haplogroup, and NC: not changed. (TIF) [file pone.0161622.s002.tif]

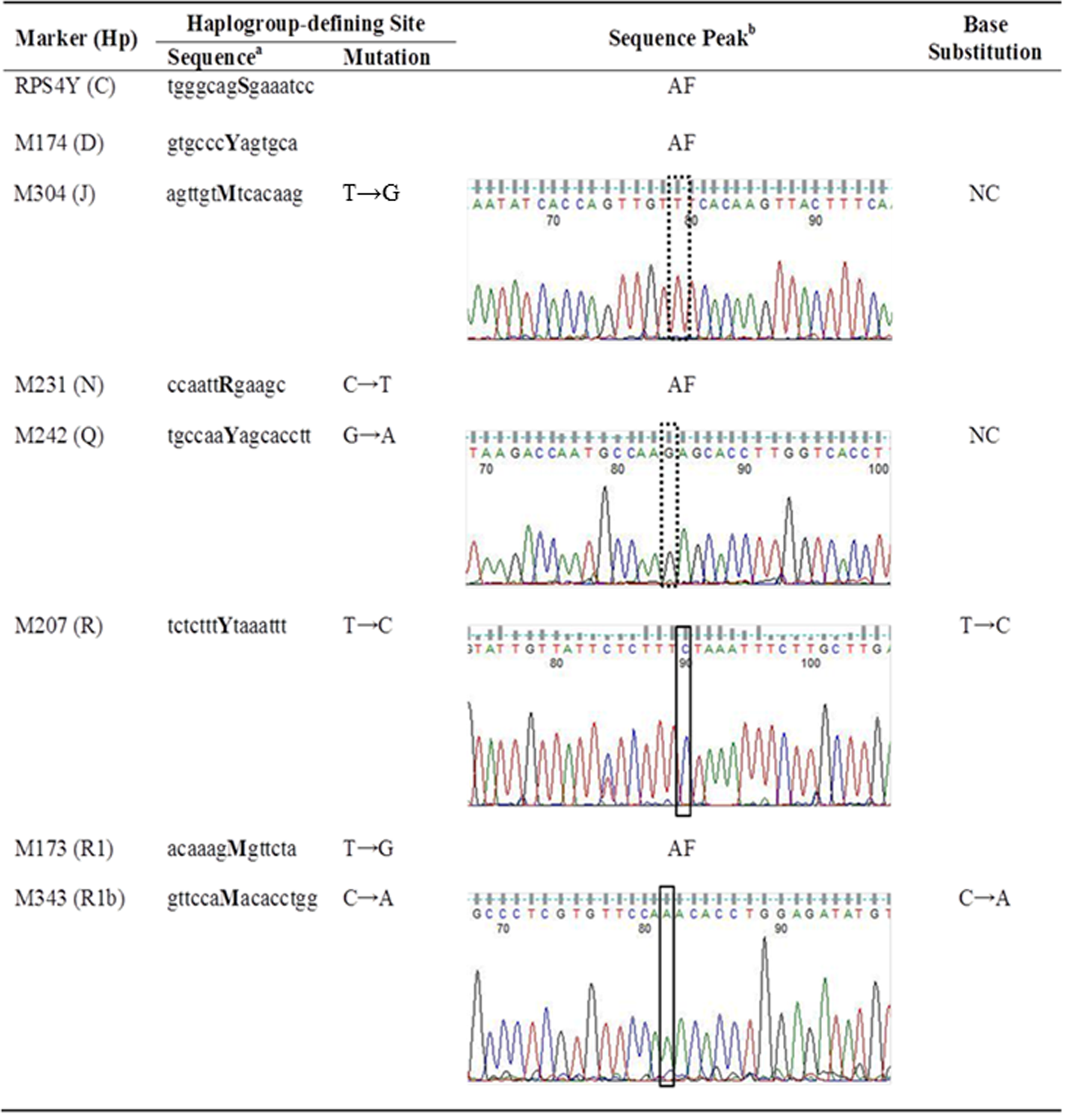

Supplement: S3 Fig — aBold characters indicate the nucleotide positions of the haplogroup-defining SNPs. bBoxes of dotted and solid lines indicate no substitution and specific mutations, respectively, in the haplogroup-defining SNPs. Hp: haplogroup, AF: failure of PCR amplification, and NC: not changed. (TIF) [file pone.0161622.s003.tif]

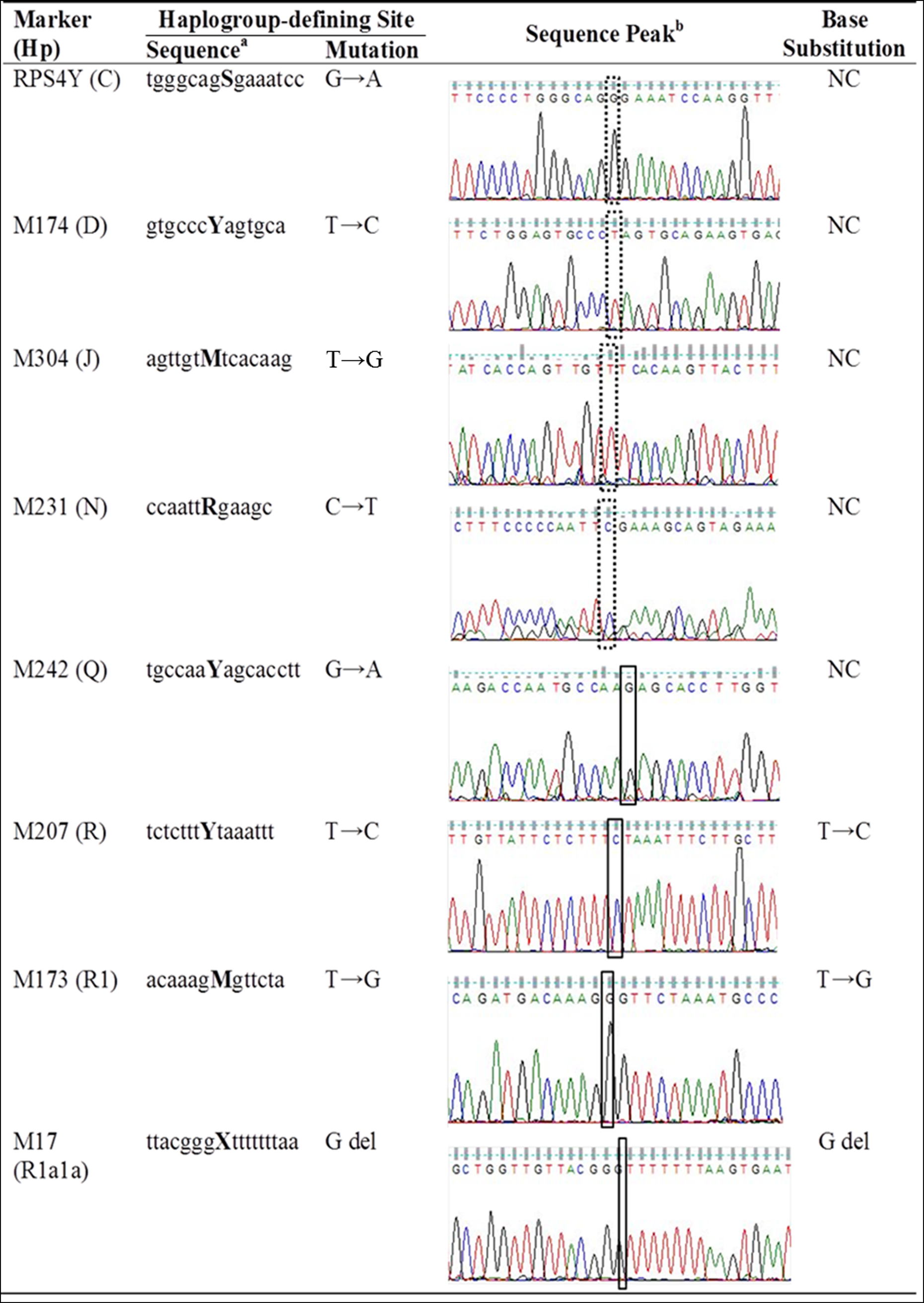

Supplement: S4 Fig — aBold characters indicate the nucleotide positions of the haplogroup-defining SNPs. bBoxes of dotted and solid lines indicate no substitution and specific mutations, respectively, in the haplogroup-defining SNPs. Hp: haplogroup, NC: not changed, and del: deletion of the base indicated. (TIF) [file pone.0161622.s004.tif]

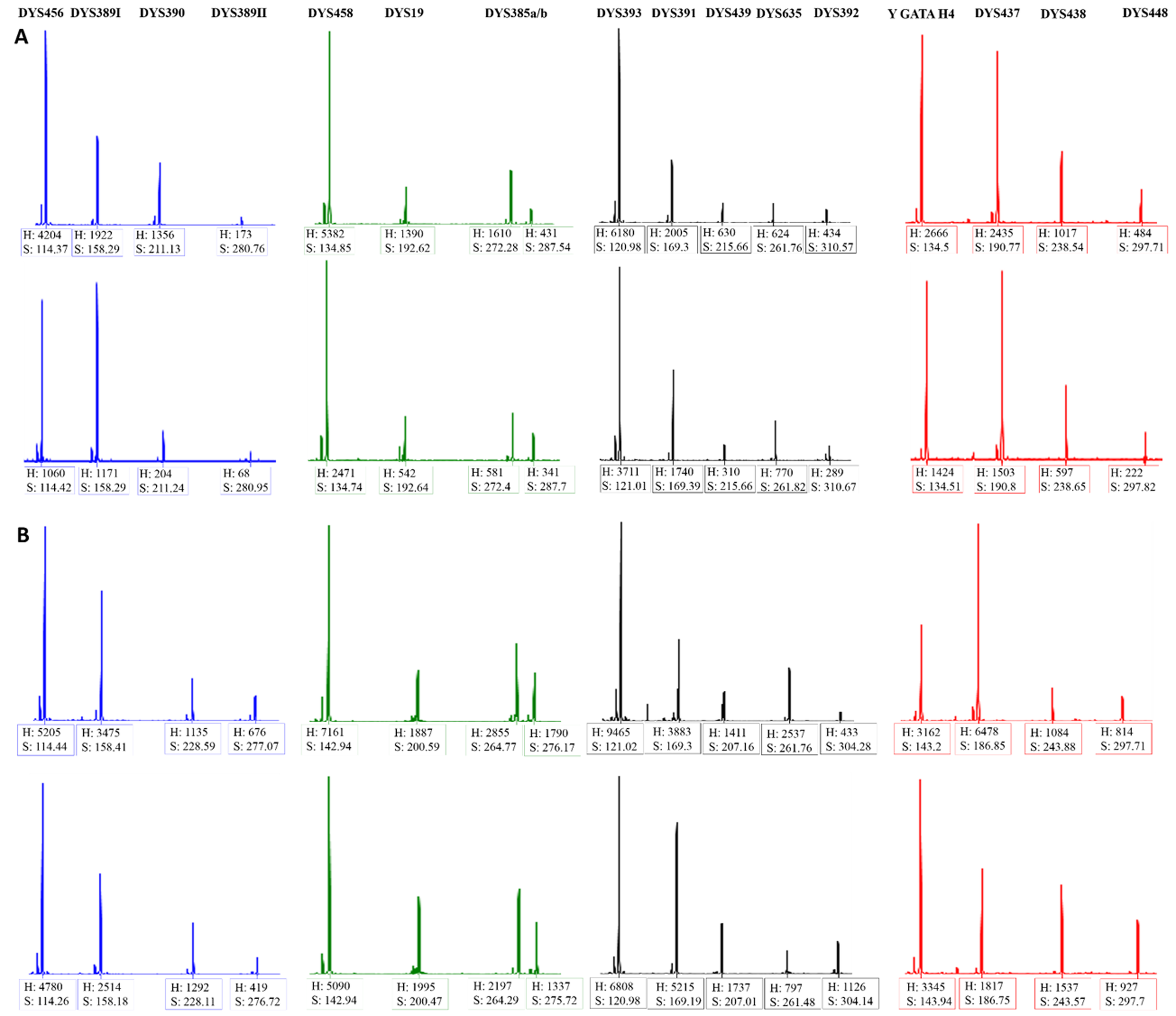

Supplement: S5 Fig — A: MN0104. B: MN0376. (TIF) [file pone.0161622.s005.tif]

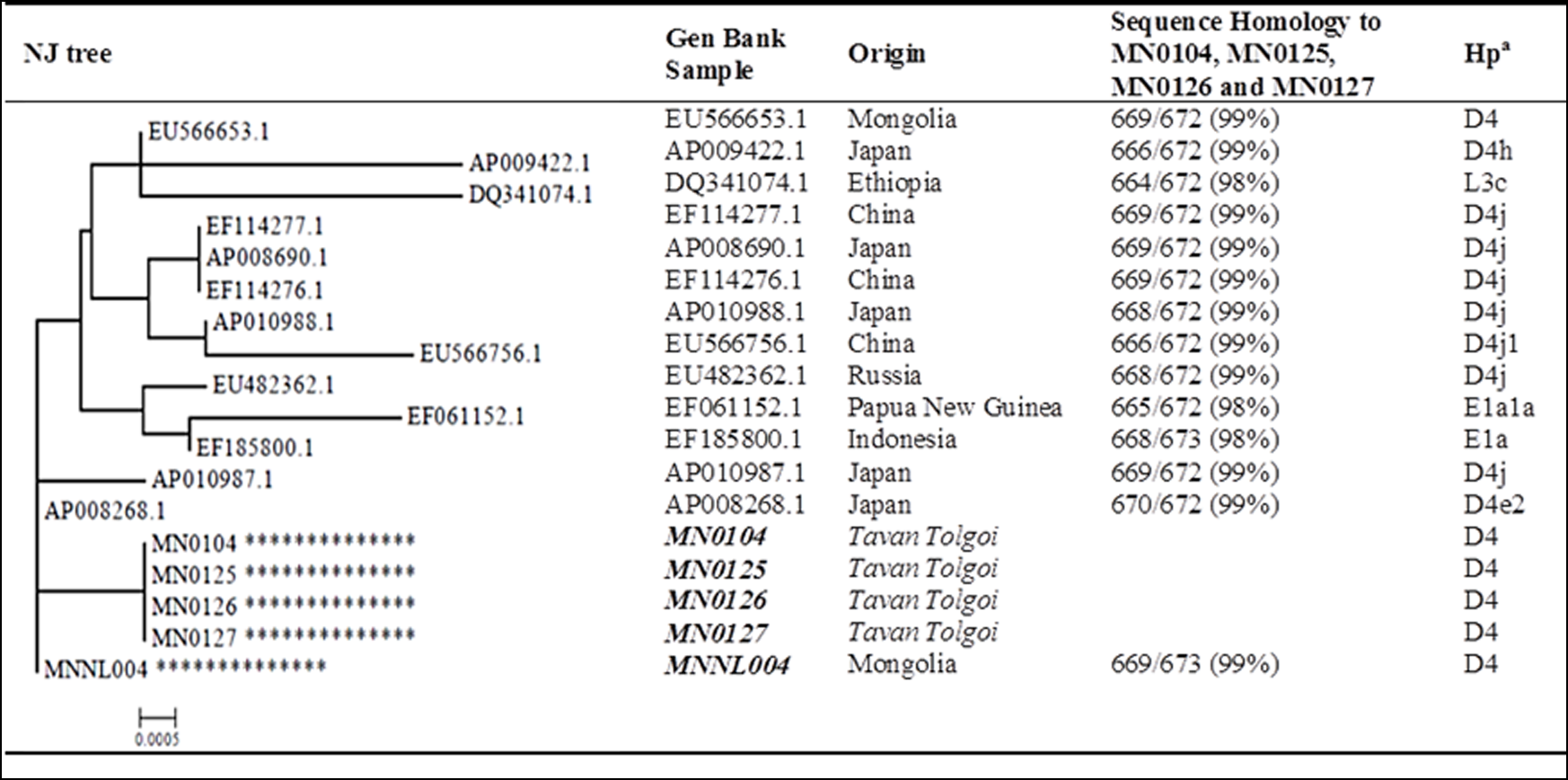

Supplement: S6 Fig — amtDNA haplogroups were determined by means of mtDNAmanager, a Web-based tool for the management and quality analysis of mtDNA sequences of control regions. (TIF) [file pone.0161622.s006.tif]

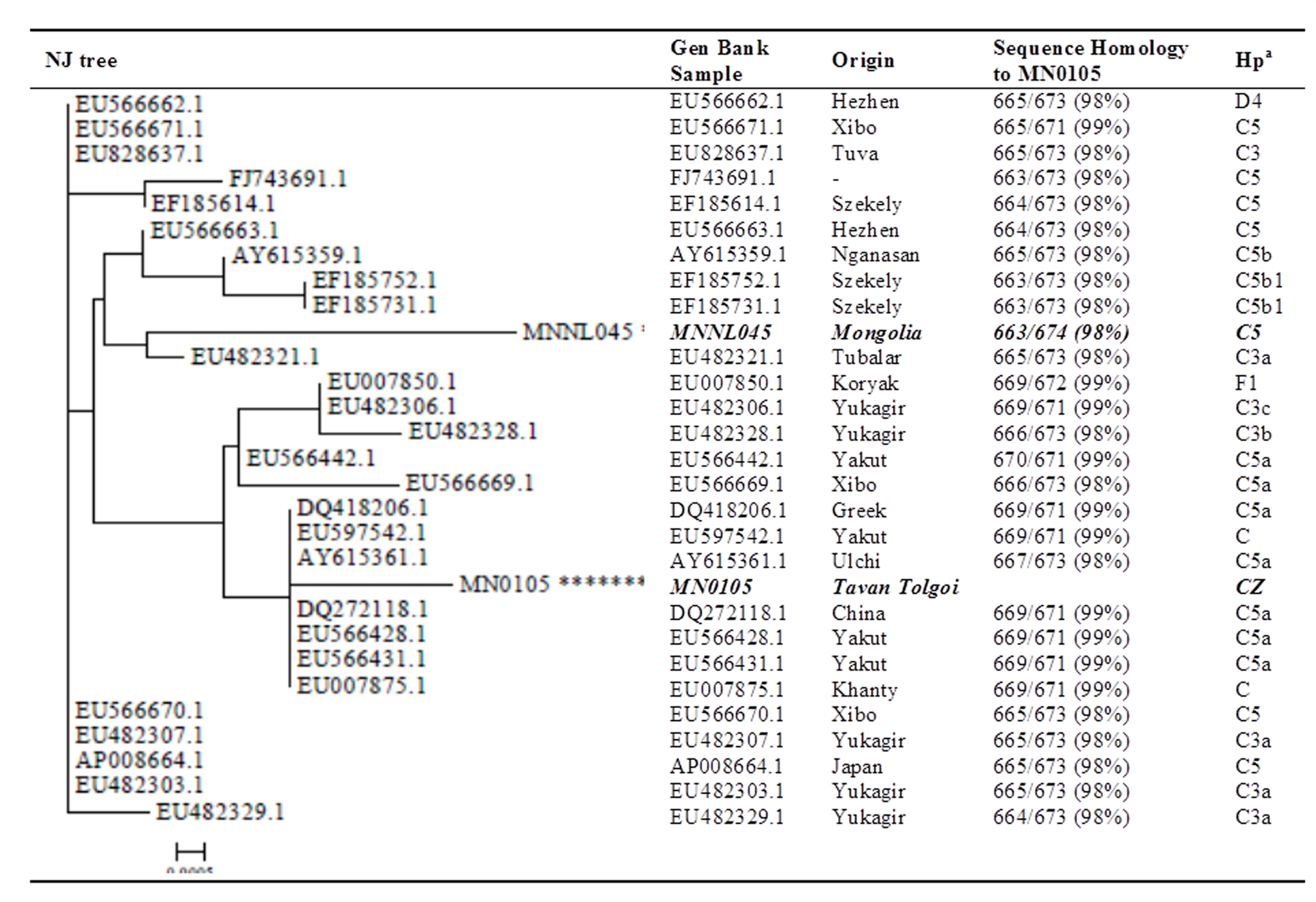

Supplement: S7 Fig — amtDNA haplogroups were determined by means of mtDNAmanager, a Web-based tool for the management and quality analysis of mtDNA sequences of control regions. Minus (-) indicates that no information is available about the population origin. (TIF) [file pone.0161622.s007.tif]

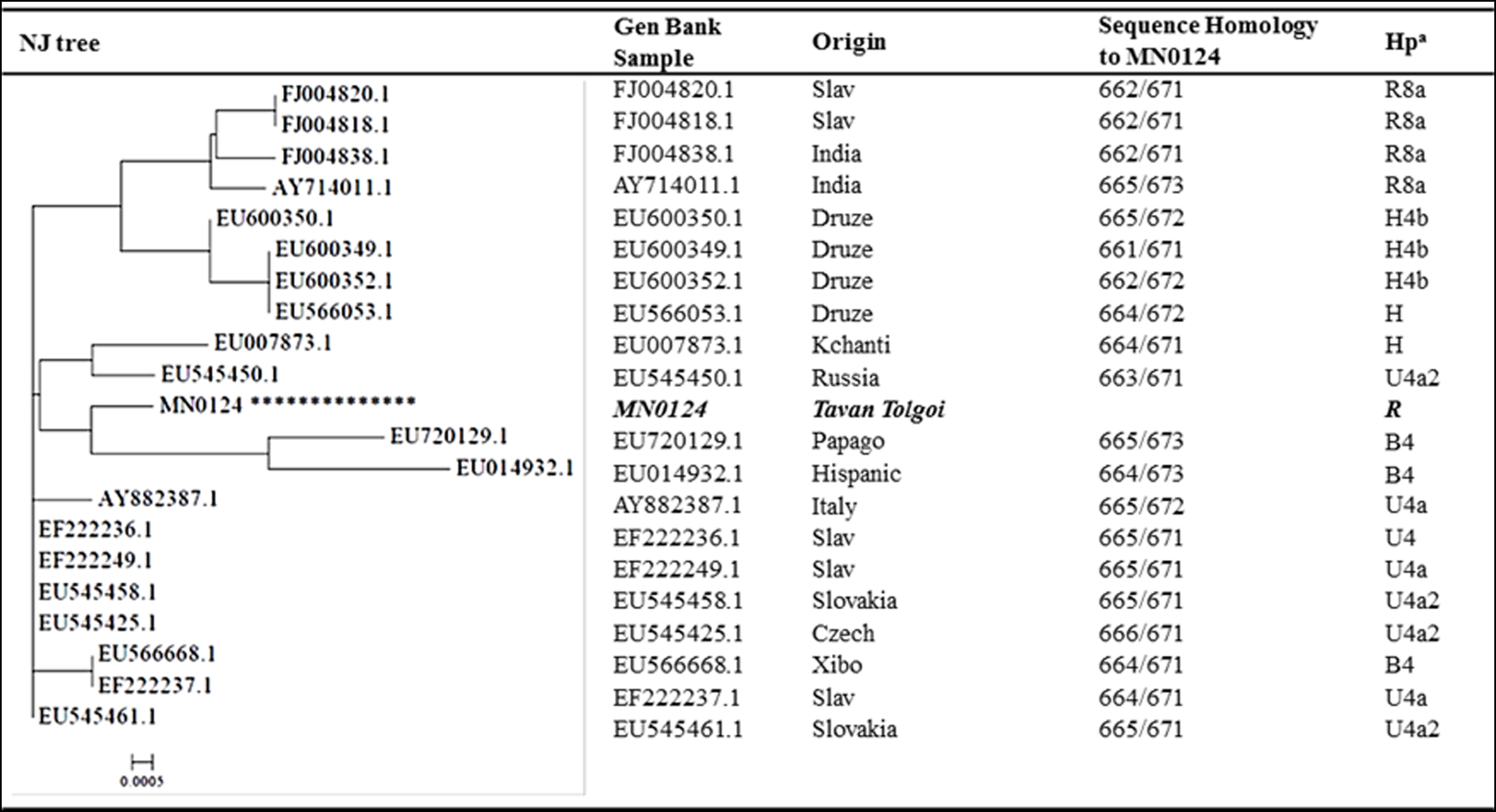

Supplement: S8 Fig — amtDNA haplogroups were determined by means of mtDNAmanager, a Web-based tool for the management and quality analysis of mtDNA sequences of control regions. (TIF) [file pone.0161622.s008.tif]

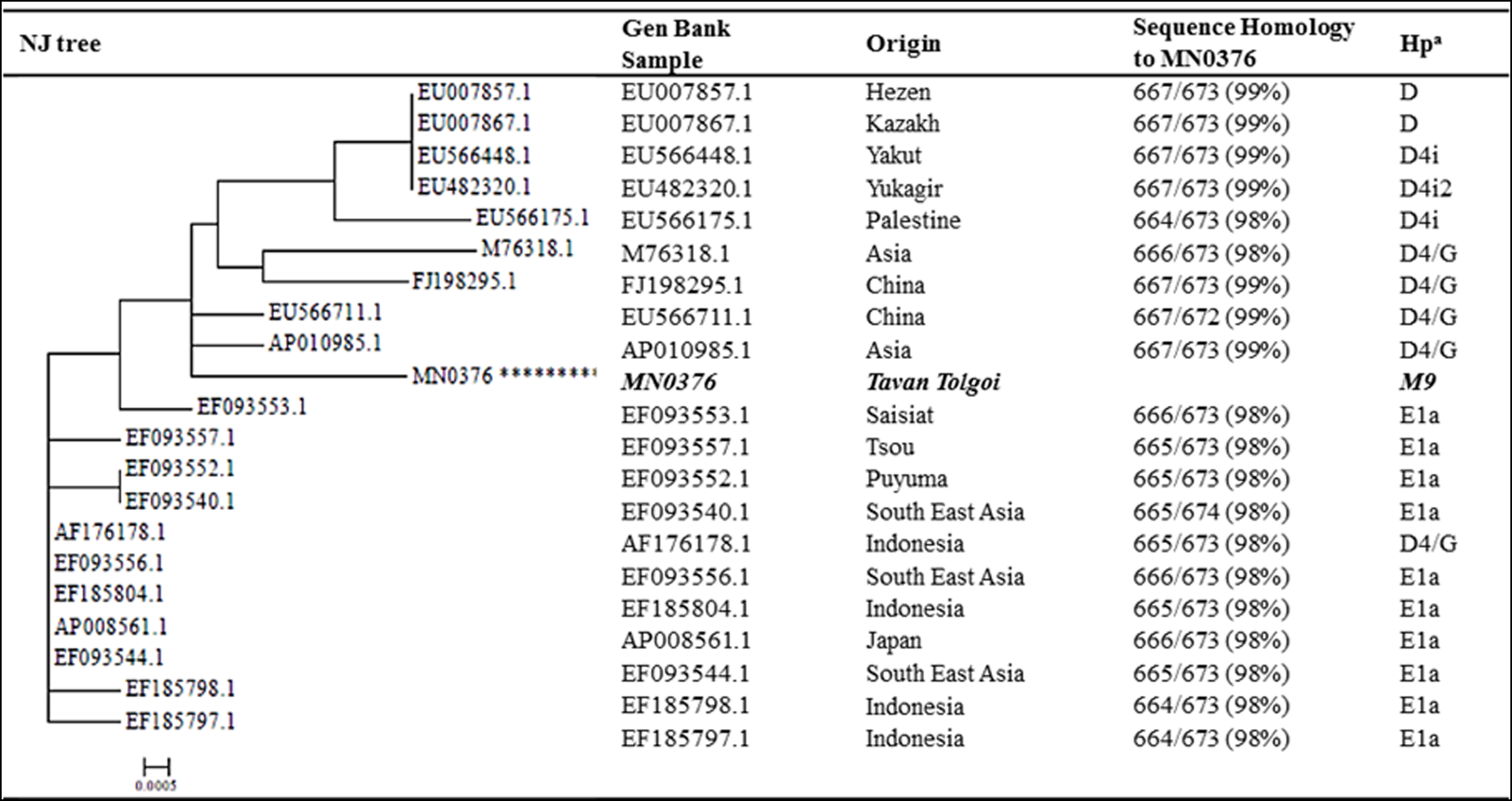

Supplement: S9 Fig — amtDNA haplogroups were determined by means of mtDNAmanager, a Web-based tool for the management and quality analysis of mtDNA sequences of control regions. (TIF) [file pone.0161622.s009.tif]

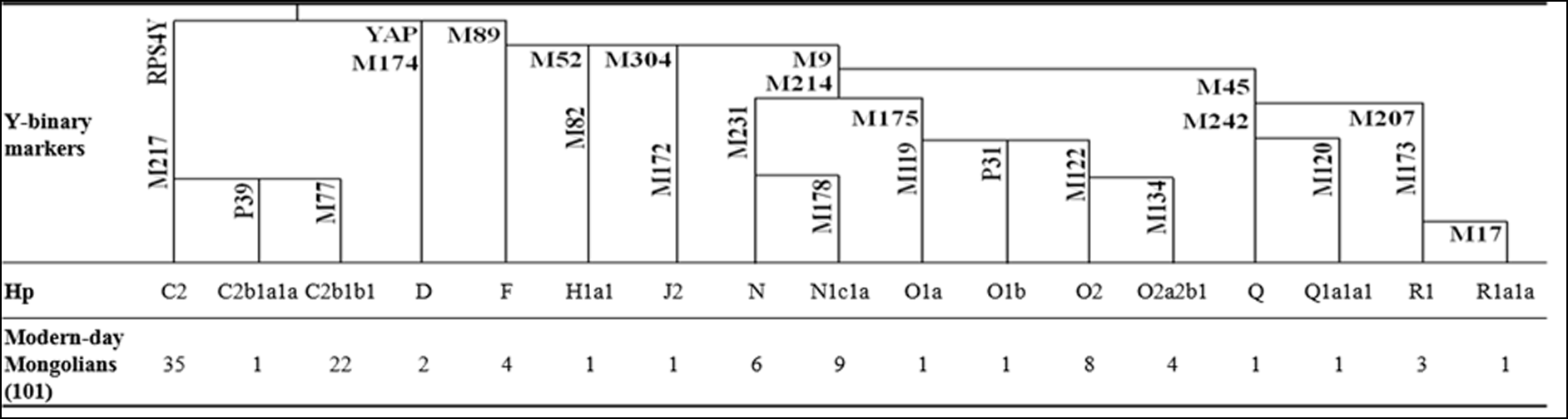

Supplement: S10 Fig — Y-haplogroups and subclades were determined using the Y-haplogroup tree of International Society of Genetic Genealogy. (TIF) [file pone.0161622.s010.tif]
