# Supplementary material for: Molecular Genealogy of a Mongol Queen’s Family and Her Possible Kinship with Genghis Khan
Source: PLoS One. 2016 Sep 14;11(9):e0161622. doi: 10.1371/journal.pone.0161622 (PMC5023095; doi:10.1371/journal.pone.0161622)
Supplement: S1 Table — aData published by Youn and colleagues [14]. According to their 14C radiocarbon dating results, the fragments of the wooden coffin of MN0127 were dated 230–540 AD. Because cinnamon usually takes 60 years to grow to a diameter of 60 cm, and therefore several hundred years to reach dimensions of 2–3 m in diameter, those authors supposed that the reason for the earlier dating of MN0127 compared with the other Golden family members could be explained by such a slow growth rate of the cinnamon. bResults of physical anthropological analyses. cThe golden rings were engraved with the falcon image that symbolizes Genghis Khan and his Borjigin clan. dMN0124 was disinterred without any noticeable artifacts except small golden earrings, according to the Mongol tradition that antenuptial children who do not own property were to be buried without any burial artifacts. ePearl and golden ornaments of boqta showing the same design and shape as those of Mongol khatuns. fIn the golden container, there were some brownish red powders that were presumed to be some kind of medicine or incense. gA bronze mirror assumed to be related to Buddhism with Sanskrit writing. hThe golden vajra (thunderbolt) that was in the hand of MN0125 and is known to be related to Buddhism. iThe golden saddle was sheathed with dragon-shaped decorations; to date, such exquisite discoveries have never been found in Mongolia. jThe golden earring that Mongolian male aristocrats used to wear in the left ear. kThe wooden coffin was made from the cinnamon plant (Cinnamomum sp.) that would have been transported from the Southern part of Asia. lThe single golden earring similar to that of MN0126 in shape and design. ND; not done. (DOCX) [file pone.0161622.s011.docx]

**S1 Table. Various characteristics of the Tavan Tolgoi graves**

| **Sample** | **GPS Location** | **^14^C Data** | **Age of Death /Sex^b^** | **aDNA Sampling** | **Human Bone** | **Animal Bone** | **Burial Artifact** |
| --- | --- | --- | --- | --- | --- | --- | --- |
| **MN0104** | N45° 05' 59,2" E112° 43' 12,8" | 1030-1270 | 25-30/ male | femur | full skeleton | full skeleton of a horse | small pieces of wooden coffin, large white pearls with golden base (called Jins) |
| **MN0105** | N45° 05' 58,9" E112° 43' 12,6" | 1030-1220 | 45-55/ female | femur | full skeleton | skeleton of a headless  horse | wooden coffin, wooden saddle sheathed in gold, two golden rings^c^, remains of clothes, ornaments of Boqta, iron stirrup |
| **MN0124** | ND | ND | teenage/ female | femur | full skeleton | some bones of a sheep | small golden earrings^d^ |
| **MN0125** | N45° 05’59,0," E112° 43’10,9" | 1150-1280 | 20-30/ female | femur | skull, femurs, sacrum, pelvis, tibias, humeri, ulnas, radius, fibula, clavicle, scapula, vertebrae, metacarpals, phalanges, metatarsals | full skeleton of a horse | wooden coffin, golden rings, golden crown, pearl and golden ornament of Boqta^e^, golden earrings, small golden container^f^, silver pot, silver bowl, human image made from jade, bronze mirrors wrapped with silk fabrics^g^, fine silk outer garment, leather boots, golden vajra^h^, harness, golden saddle sheathed with dragon image^i^, various golden and silver goods |
| **MN0126** | E45° 05’58,7" N112° 43’11,1" | ND | 20-25/ male | femur | skull, femurs, humeri, ulnas, radius, tibias, metatarsals, phalanges, metacarpals, ribs, vertebrae | vertebrae of a horse | wooden coffin, saddle tip made by bone, a single golden earring^j^, copper and iron goods |
| **MN0127** | N45° 05’58,9" E112° 43’10,7" | 230-540^a^ | 25-30/ male | femur/ humerus | skull, ulna, fragments of femur, tarsals, metatarsals, phalanges | full skeleton of a horse | wooden coffin^k^, a single golden earring^l^, clothes, a petal golden ochir, jade belt decoration inlaid with square turquoise, 7-petal decoration made of bones |
| **MN0376** | N45° 05’55,7" E112° 42’47,1" | ND | 40-45/ male | femur | full skeleton | sheep’s shoulder blades, ribs, ankles, vertebrae, tibias, a horse head | horse harness, stirrup, birch-bark arrow quiver with four arrowheads inside |
