# Supplementary material for: Molecular Genealogy of a Mongol Queen’s Family and Her Possible Kinship with Genghis Khan
Source: PLoS One. 2016 Sep 14;11(9):e0161622. doi: 10.1371/journal.pone.0161622 (PMC5023095; doi:10.1371/journal.pone.0161622)
Supplement: S2 Table — aEstimation of cranial metric traits was conducted according to the method proposed by Martin [63]. Minus (-) indicates inability to estimate cranial metric traits due to partial breakage of a skull. Data from MN0125 and MN0127 are not available because their skulls were badly broken and did not allow for estimation of cranial metric traits. (DOCX) [file pone.0161622.s012.docx]

**S2 Table. Osteometric parameters of skulls of the Tavan Tolgoi bodies**

| **Cranial Metric Traits^a^** | | **MN0104** | **MN0105** | **MN0126** | **MN0124** | **MN0376** |
| --- | --- | --- | --- | --- | --- | --- |
| M1 | Maximum cranial length | 190 | 167 | - | 171 | 162 |
| M8 | Maximum cranial breadth | 152 | 152 | - | 147 | 155 |
| M17 | Basion-bregma height | 125 | 123 | - | - | - |
| M5 | Basion-nasion length | 100 | 97 | - | - | - |
| M9 | Least frontal breadth | 101 | 96 | 95 | 85 | 98 |
| M11 | Biauricular breadth | 133 | 136 | - | 115 | - |
| M12 | Biasterionic breadth | 112 | 113 | 108 | 104 | - |
| M7 | Frontal sagittal chord | 122 | 106 | 111 | 109 | 99 |
| M30 | Bregma-lambda chord | 103 | 104 | 104 | 116 | 109 |
| M31 | Lambda-opisthion chord | 92 | 95 | 99 | - | - |
| M23 | Horizontal circumference | 540 | - | - | 500 | - |
| M24 | Transverse arc | 338 | 316 | - | 328 | - |
| M25 | Total sagittal arc | 145 | 121 | 126 | 128 | 121 |
| M26 | Frontal sagittal arc | 110 | 116 | 117 | 132 | 122 |
| M27 | Parietal sagittal arc | 120 | 110 | 129 | - | - |
| M45 | Bizygomatic breadth | 141 | 141 | - | 120 | 143 |
| M40 | Basion-prosthion length | 96 | 86 | - | - | - |
| M48 | Upper facial height | 77 | 69 | 67 | 60 | 72 |
| M47 | Nasomental length | 132 | - | - | - | - |
| M43 | Outer biorbital breadth | 110 | 108 | 102 | 94 | 112 |
| M46 | Bimaxillary breadth | 100 | 104 | - | 92 | 99 |
| M55 | Nasal height | 59 | 51 | 52 | 46 | 54 |
| M54 | Nasal breadth | 25 | 29 | 24 | 28 | 26 |
| M51 | Orbital breadth (R) | 43 | 43 | 38 | 36 | 42 |
| M52 | Orbital height (R) | 37 | 35 | 35 | 34 | 38 |
| M8/1 | Longer-breadth index in cranial | 80 | 91,02 | - | 85,96 | 95,68 |
| M17/1 | Longer-height index in cranial | 65,79 | 73,65 | - | - | - |
| M17/8 | Breadth-height index in cranial | 82,24 | 80,92 | - | - | - |
| M47/45 | Total facial index (Kollmann) | 93,62 | - | - | - | - |
| M48/45 | Upper facial index (Kollmann) | 54,61 | 48,94 | - | 50 | 50,35 |
| M47/46 | Facial index (Virchow) | 132 | - | - | - | - |
| M48/46 | Upper facial index (Virchow) | 77 | 66,35 | - | 65,22 | 72,73 |
| M52/51 | Orbital index (R) | 86,04 | 81,39 | 92,10 | 94,44 | 90,48 |
| M54/55 | Nasal index | 42,37 | 56,86 | 46,15 | 60,86 | 48,15 |
| Height (cm) | | 169.8 | 165.6 |  |  |  |
| Weight (kg) | | 78.1 | 68.1 |  |  |  |
