# Supplementary material for: Molecular Genealogy of a Mongol Queen’s Family and Her Possible Kinship with Genghis Khan
Source: PLoS One. 2016 Sep 14;11(9):e0161622. doi: 10.1371/journal.pone.0161622 (PMC5023095; doi:10.1371/journal.pone.0161622)
Supplement: S3 Table — aNucleotide sequences of amplicons were obtained using PCR-directed sequencing of mtDNA HVR1. Minus (-) and blank indicate failure of PCR amplification and failure to clone PCR products, respectively. PCR-directed sequencing data were identical to the sequencing data from all active and inactive UDG–treated clones in their consensus sequences. Con: consensus sequence. (DOCX) [file pone.0161622.s013.docx]

**S3 Table. Among the Tavan Tolgoi bodies (MN0104, MN0125, and MN0126), comparison of HVR1 nucleotide sequences between direct PCR products and clones obtained after treatment with either active or inactive uracil DNA glycosylase (UDG)**

| **UDG Treatment** | **MN0104** | | | | |  | **MN0125** | | | | | | |  | **MN0126** | | | |
| --- | --- | --- | --- | --- | --- | --- | --- | --- | --- | --- | --- | --- | --- | --- | --- | --- | --- | --- |
|  | **Clone No** | **16071G** | **16223G** | **16311C** | **16362C** |  | **16070A** | **16171G** | **16223G** | **16277A** | **16311C** | **16340A** | **16362C** |  | **16171G** | **16223G** | **16311C** | **16362C** |
| Active | 1 | G | T | C | C |  | A | G | T | A | T | A | C |  | G | T | T | C |
|  | 2 | G | T | C | C |  | A | G | T | A | T | A | C |  | G | T | T | C |
|  | 3 | G | T | C | C |  | A | G | T | A | T | A | C |  | G | T | T | C |
|  | 4 | G | T | C | C |  | A | G | T | A | T | **G** | C |  | G | T | T | C |
|  | 5 | G | T | C | C |  | A | G | T | A | T | A | C |  |  |  |  |  |
|  | 6 | G | T | C | C |  | A | G | T | A | T | A | C |  |  |  |  |  |
|  | 7 | G | T | C | C |  | A | G | T | T | T | A | C |  |  |  |  |  |
|  | 8 | G | T | C | C |  | A | G | T | A | T | A | C |  |  |  |  |  |
|  | 9 | G | T | C | C |  | A | G | T | A | T | A | C |  |  |  |  |  |
|  | 10 | G | T | C | C |  | A | G | T | A | T | A | C |  |  |  |  |  |
|  | **Con** | **G** | **T** | **C** | **C** |  | **A** | **G** | **T** | **A** | **T** | **A** | **C** |  | **G** | **T** | **T** | **C** |
| Inactive | 1 | G | T | C | C |  | A | G | T | A | T | A | C |  | G | T | T | C |
|  | 2 | G | T | C | C |  | A | G | T | A | T | A | C |  | G | T | T | C |
|  | 3 | G | T | C | C |  | A | G | T | A | T | A | C |  | G | T | T | C |
|  | 4 | G | T | C | C |  | A | G | T | A | T | A | C |  | G | T | T | C |
|  | 5 | G | T | C | C |  | G | G | T | A | T | A | C |  | G | T | T | C |
|  | 6 | - | T | C | C |  | A | G | T | A | T | A | C |  |  |  |  |  |
|  | 7 | - | T | C | C |  | A | G | T | A | T | A | C |  |  |  |  |  |
|  | 8 | - | T | C | C |  | A | G | T | A | T | A | C |  |  |  |  |  |
|  | 9 | - | T | C | C |  | A | G | T | A | T | A | C |  |  |  |  |  |
|  | 10 | - | T | C | C |  | A | G | T | A | T | A | C |  |  |  |  |  |
|  | **Con** | **G** | **T** | **C** | **C** |  | **A** | **G** | **T** | **A** | **T** | **A** | **C** |  | **G** | **T** | **T** | **C** |
| Amplicon^a^ | **Con** | **G** | **T** | **C** | **C** |  | **A** | **G** | **T** | **A** | **T** | **A** | **C** |  | **G** | **T** | **T** | **C** |
