# Supplementary material for: Molecular Genealogy of a Mongol Queen’s Family and Her Possible Kinship with Genghis Khan
Source: PLoS One. 2016 Sep 14;11(9):e0161622. doi: 10.1371/journal.pone.0161622 (PMC5023095; doi:10.1371/journal.pone.0161622)
Supplement: S4 Table — aNucleotide sequences of amplicons were obtained by PCR-directed sequencing of mtDNA HVR1. Minus (-) and blank indicate failure of PCR amplification and failure to clone PCR products, respectively. All PCR-directed sequencing data were identical to the sequencing data from active UDG-treated clones in their consensus sequences, except for the C→T substitution (the box in the table) in the 5 clones derived from inactive UDG-treated PCR products of MN0376. A solid box indicates the substituted base; base T was converted to base C after incubation with active UDG, meaning that aDNA from MN0376 had postmortem damage (C→T) and the damaged base was restored to the authentic base C by UDG. Con: consensus sequence. (DOCX) [file pone.0161622.s014.docx]

**S4 Table**

| **UDG treatment** |  | **MN0105** | | | | |  | **MN0124** | | |  | **MN0376** | | | | |
| --- | --- | --- | --- | --- | --- | --- | --- | --- | --- | --- | --- | --- | --- | --- | --- | --- |
|  | **Clone No** | **16093C** | **16223T** | **16261T** | **16288C** | **16298C** |  | **16168C** | **16217C** | **16271T** |  | **16223T** | **16234T** | **16250C** | **16299G** | **16362C** |
| Active | 1 | - | T | T | C | C |  | A | C | T |  | T | T | C | G | C |
|  | 2 | - | T | T | C | C |  | C | C | T |  | T | T | C | G | C |
|  | 3 | - | T | T | C | C |  | C | C | C |  | T | T | C | G | C |
|  | 4 | - | T | T | C | C |  | C | C | T |  | T | T | C | G | C |
|  | 5 | - | T | T | C | C |  | C | C | T |  | T | T | C | G | C |
|  | 6 | - |  |  |  |  |  | C | C | T |  |  |  |  |  |  |
|  | 7 | - |  |  |  |  |  | C | C | T |  |  |  |  |  |  |
|  | 8 | - |  |  |  |  |  | C | C | T |  |  |  |  |  |  |
|  | 9 | - |  |  |  |  |  | C | C | T |  |  |  |  |  |  |
|  | 10 | - |  |  |  |  |  | C | C | T |  |  |  |  |  |  |
|  | **Con** |  | **T** | **T** | **C** | **C** |  | **C** | **C** | **T** |  | **T** | **T** | **C** | **G** | **C** |
| Inactive | 1 |  | T | T | C | C |  | C | C | T |  | T | T | T | G | C |
|  | 2 |  | T | T | C | C |  | C | C | T |  | T | T | T | G | C |
|  | 3 |  | T | T | C | C |  | C | C | T |  | T | T | T | G | C |
|  | 4 |  | T | T | C | C |  | C | C | T |  | T | T | T | G | C |
|  | 5 |  | T | T | C | C |  | C | C | T |  | T | T | T | G | C |
|  | 6 |  |  |  |  |  |  | C | C | T |  |  |  |  |  |  |
|  | 7 |  |  |  |  |  |  | C | C | T |  |  |  |  |  |  |
|  | 8 |  |  |  |  |  |  | C | C | T |  |  |  |  |  |  |
|  | 9 |  |  |  |  |  |  | C | C | T |  |  |  |  |  |  |
|  | 10 |  |  |  |  |  |  | C | C | T |  |  |  |  |  |  |
|  | **Con** |  | **T** | **T** | **C** | **C** |  | **C** | **C** | **T** |  | **T** | **T** | **T** | **G** | **C** |
| Amplicon^a^ | **Con** | **C** | **T** | **T** | **C** | **C** |  | **C** | **C** | **T** |  | **T** | **T** | **C** | **G** | **C** |
