# Supplementary material for: Molecular Genealogy of a Mongol Queen’s Family and Her Possible Kinship with Genghis Khan
Source: PLoS One. 2016 Sep 14;11(9):e0161622. doi: 10.1371/journal.pone.0161622 (PMC5023095; doi:10.1371/journal.pone.0161622)
Supplement: S5 Table — aNomenclature of mtDNA haplogroups is based on the updated tree of mtDNA haplogroup. Numbers indicate nucleotide positions based on the revised Cambridge Reference Sequence (rCRS). bPrimer set used for amplification of HVR1 in clones obtained after treatment with UDG. cPrimer set used for amplification of haplogroup (M, N and Z)-defining SNP sites. dPrimer set used for amplification of haplofroup (CZ and G)-defining SNP sites. eNomenclature of Y-haplogroups is based on the revised haplogroup tree. (DOCX) [file pone.0161622.s015.docx]

**S5 Table. Primers for identification of haplogroups of mtDNA and the Y-chromosome**

| **Name of haplogroup** | | **Sequence (5'→3')** | | **Size (bp)** | **Reference** |
| --- | --- | --- | --- | --- | --- |
|  |  | **Forward** | **Reverse** |  |  |
| **the site mutated^a^** | | | | |  |
| HVR1 | Outer (15971-16410) | TTAACTCCACCATTAGCACC | GAGGATGGTGGTCAAGGGAC | 440 | [66] |
|  | Nested (15977-16399) | CCACCATTAGCACCCAAAGC | TCAAGGGACCCCTATCTGA | 423 | [18] |
|  | 16097-16410^b^ | TACATTACTGCCAGCCAC | GAGGATGGTGGTCAAGGGAC | 314 | This study |
| HVR2 | Outer(15-389) | CACCCTATTAACCACTCACG | CTGGTTAGGCTGGTGTTAGG | 375 | [66] |
| M or N (10398)  Z (10325) | Nested (29-381) | CTCACGGGAGCTCTCCAT | GCTGGTGTTAGGGTTCTTTG | 353 | [18] |
|  | 10130-10484**^c^** | ACCACAACTCAACGGCTACA | TAAATGAGGGGCATTTGG | 355 | [23] |
| M7 (9824) | 9744-9910 | AGATACTTCGAGTCTCC | AGCCAAGTGATGTTTGG | 167 | [23] |
| M9 (3393) | 3325-3476 | CCTACTCCTCATTGTACC | AGTTTTATGGCGTCAGCG | 152 | This study |
| CZ (4715)  G (4833) | 4646-4900**^d^** | TATTTCCTCACGCAAGC | GGTATATGATTGAGATGG | 255 | This study |
| D (5178) | 5047-5313 | TTCTACCGTACAACCCTAACATAAC | GTGAATTCTTCGATAATGGC | 267 | [23] |
| D4 (3010) | 2934-3079 | GGGATAACAGCGCAATCC | GTCTGAACTCAGATCACG | 146 | [23] |
| N9 (5417) | 5337-5495 | CCTCTACTTCTACCTACG | AAAGGGGAGATAGGTAGG | 159 | [23] |
| A (663) | 587-741 | AGCTTACCTCCTCAAAGC | GTGGTGATTTAGAGGGTG | 155 | [23] |
| R (12705) | 12671-12797 | TTGTTCGTTACATGGTCC | GCAAGAAGGATATAATTCC | 127 | [23] |
| B (8291-8299) | 8191-8389 | CAAACCACAGTTTCATGC | ACGGTAGTATTTAGTTGGG | 199 | [23] |
|  | | | | | |
| **Y chromosome^e^** | | | | | |
| M175 (O) | Outer | AAAATAGTACCCAAATCAACTCA | TTCTCTTGCAGCATTTTCAGTT | 231 | This study |
|  | Nested | GCACATGCCTTCTCACTTCT | TTCAGTTAGCCTTGATTGACTGT | 164 | [18] |
| RPS4Y_711_ (C) | Outer | CAGGGCAATAAACCTTGGAT | CCACAAGGGGGAAAAAACAC | 188 | [18] |
|  | Nested | GGGCAATAAACCTTGGATTT | CATTAAGAAACGAGAATTCACTG | 167 | [18] |
| M174 (D) | Outer | CACTGTGCGTTCTTCTCCGTCAC | TGACTAGAAGGTCCTGGAGATGC | 217 | [18] |
|  | Nested | CTCCGTCACAGCAAAAATGTAC | CCCATCTTGCAAGGAAAAGT | 162 | [18] |
| M231 (N) | Outer | GGAAAATGTGGGCTCGTTT | GAATGGTGGCCAGAGTCTTTCAC | 207 | [18] |
|  | Nested | GGCTCGTTTTAATTATATTCATAT | GGCCAGAGTCTTTCACATCAT | 190 | [18] |
| M207 (R) | Outer | ACTATGGGGCAAATGTAAGT | GCTGTTCGCTGCTACGAAT | 162 | [18] |
|  | Nested | GGCAAATGTAAGTCAAGCAAGAA | CGCTGCTACGAATCTTTAATCTTA | 149 | [18] |
| M242 (Q) | Outer | TCTACGGCATAGAAAGTTTGTG | GCTTTAAGGGCTTTCAGCAT | 168 | [18] |
|  | Nested | TGTGCAAAAAGGTGACCAA | GGCTTTCAGCATAATACCTTAC | 142 | [18] |
| M304 (J) | Outer | GGTATTGGGGTAGGCAAAGA | CCTTCAGGCTTCTAGCTTCATC | 207 | [18] |
|  | Nested | TGGGGTAGGCAAAGAAAAG | GGCTTCTAGCTTCATCTGCATTGT | 196 | [18] |
| M173 (R1) | Outer | CTTACAATTCAAGGGCATTTAG | AAAAGTCAACAGGGCTTTG | 230 | [18] |
|  | Nested | CAATTCAAGGGCATTTAGAA | CTTTACCTTCACAGCCTTCA | 165 | [18] |
| M343 (R1b) | Outer | AGGAGGCGGTGTCTGATT | ACCCCCACATATCTCCAGG | 153 | [18] |
|  | Nested | AAGGCTCAGGGTATTGGTT | CCCACATATCTCCAGGTG | 127 | [18] |
| M17 (R1a1a) | Outer | GGTCAAAAGAAGGCGTAGATAC | CAAAAATAGTTTGGCCACTTAAC | 174 | [18] |
|  | Nested | TTTGGCCTTGTCTTGAAGTG | ACTTAACAAACCCCAAAATTCAC | 134 | This study |
