# Supplementary material for: Molecular Genealogy of a Mongol Queen’s Family and Her Possible Kinship with Genghis Khan
Source: PLoS One. 2016 Sep 14;11(9):e0161622. doi: 10.1371/journal.pone.0161622 (PMC5023095; doi:10.1371/journal.pone.0161622)
Supplement: S6 Table — aGe. Hp: Genotyped Haplogroup, Haplogroup previously determined by Y-SNP analysis in the literature indicated. bYHRD: Y-Chromosome STR Haplotype Reference Database. cPre. Hp: Predicted Haplogroup, Haplogroup predicted using the Yfiler and PowerPlex Y of YHRD, based on the Y-STR profiles. Hp: haplogroup, NM: No match, ND: Not done, and NDT: Not determined. (DOCX) [file pone.0161622.s016.docx]

**S6 Table. Modern-day individuals with the Y-STR profile matching that of the Tavan Tolgoi body (MN0104)**

| **Sample** | **Population** | **Ge. Hp^a^** | **Y-STR marker** | | | | | | | | | | | | | | | |  | **YHRD^b^** | | **Reference** |
| --- | --- | --- | --- | --- | --- | --- | --- | --- | --- | --- | --- | --- | --- | --- | --- | --- | --- | --- | --- | --- | --- | --- |
|  |  |  | **DYS 19** | **DYS 385** | **DYS 389I** | **DYS 389II** | **DYS 390** | **DYS 391** | **DYS 392** | **DYS 393** | **DYS 437** | **DYS 438** | **DYS 439** | **DYS 448** | **DYS 456** | **DYS 458** | **DYS 635** | **YGATA H4** |  | **Pre. Hp^c^** | **Probability (%)** |  |
| MN0104 | Mongolian | R1b-M343 | 14 | 13/17 | 13 | 30 | 22 | 11 | 13 | 13 | 15 | 10 | 12 | 20 | 15 | 15 | 23 | 11 |  | R1b | 66.7 | This study |
| Kalmyk 73 | Kalmyk | ND | 14 | 13/17 | 13 | 30 | 22 | 11 | 13 | 13 | 15 | 10 | 12 | 20 | 15 | 15 | 23 | 11 |  | R1b | 66.7 | [33], YHRD |
|  | Hui(Chinese) | ND | 14 | 13/17 | 13 | 30 | 22 | 11 | 13 | 13 | 15 | 10 | 12 | 20 | 15 | 15 | 23 | 11 |  | R1b | 66.7 | YHRD |
| Russian | Russian | R1*-M173 | 14 | 13-17 | 13 | 30 | 22 | 11 | 13 | 13 | 15 | 10 | 13 | 20 | 15 | 15 | 23 | 11 |  | R1b | 66.7 | [34] |
| 26 | Uzbek | R1b1a*-P297 | 14 | 13-17 | 13 | 31 | 22 | 11 | 13 | 13 | 15 | 10 | 12 | 20 | 15 | 16 | 23 | 11 |  | NM | - | [35] |
| 134 | Tajik | R1b1a*-P297 | 14 | 13-17 | 13 | 31 | 22 | 11 | 13 | 13 | 15 | 10 | 12 | 20 | 15 | 16 | 23 | 11 |  | NM | -∙ | [35] |
|  | Uzbek | ND | 14 | 13/17 | 13 | 30 | 22 | 11 | 13 | 13 | 15 | 10 | 12 | - | - | - | - | - |  | R1b | 66.7 | YHRD |
