# Supplementary material for: Molecular Genealogy of a Mongol Queen’s Family and Her Possible Kinship with Genghis Khan
Source: PLoS One. 2016 Sep 14;11(9):e0161622. doi: 10.1371/journal.pone.0161622 (PMC5023095; doi:10.1371/journal.pone.0161622)
Supplement: S7 Table — aGe. Hp: Genotyped Haplogroup, Haplogroup previously determined by Y-SNP analysis in the literature indicated. bYHRD: Y-Chromosome STR Haplotype Reference Database. cPre. Hp: Predicted Haplogroup, Haplogroup predicted using the Yfiler and PowerPlex Y of YHRD, based on the Y-STR profiles. Hp: haplogroup, NM: No match, ND: Not done, and NDT: Not determined. (DOCX) [file pone.0161622.s017.docx]

**S7 Table. Modern-day individuals with the Y-STR profile matching that of the Tavan Tolgoi body (MN0376)**

| **Sample** | **Population** | **Ge. Hp^a^** | **Y-STR marker** | | | | | | | | | | | | | | | |  | **YHRD^b^** | | **Reference** |
| --- | --- | --- | --- | --- | --- | --- | --- | --- | --- | --- | --- | --- | --- | --- | --- | --- | --- | --- | --- | --- | --- | --- |
|  |  |  | **DYS 19** | **DYS 385** | **DYS 389I** | **DYS 389II** | **DYS 390** | **DYS 391** | **DYS 392** | **DYS 393** | **DYS 437** | **DYS 438** | **DYS 439** | **DYS 448** | **DYS 456** | **DYS 458** | **DYS 635** | **YGATA H4** |  | **Pre. Hp^c^** | **Probability (%)** |  |
| MN0376 | Mongolian | R1a1a-M17 | 16 | 11/14 | 13 | 29 | 26 | 11 | 11 | 13 | 14 | 11 | 10 | 20 | 15 | 17 | 23 | 13 |  | NM | NDT | This study |
| Tverskaja | Russian | R1a1a-M17 | 16 | 11/14 | 13 | 31 | 26 | 11 | 11 | 13 | 14 | 11 | 10 | 20 | 16 | 16 | 23 | 13 |  | R1a | 100.0 | [34] |
| Ht52 | Hui (Chinese) | ND | 16 | 11/14 | 13 | 31 | 25 | 11 | 11 | 13 | 14 | 11 | 10 | 20 | 15 | 16 | 23 | 13 |  | R1a | 100.0 | [36] |
| 109 | Croatian | ND | 16 | 11/14 | 13 | 30 | 26 | 11 | 11 | 13 | 14 | 11 | 11 | 20 | 15 | 15 | 23 | 13 |  | R1a | 100.0 | [37] |
| 191 | Pashtun (Afghan) | R1a1a-M17 | 16 | 11/14 | 13 | 29 | 24 | 11 | 11 | 13 | 14 | 11 | 10 | 20 | 15 | 15 | 24 | 13 |  | R1a | 100.0 | [35] |
| Afg179 | Pashtun (Afghan) | R1a1a* -M198 | 16 | 11/14 | 13 | 29 | 24 | 11 | 11 | 13 | 14 | 11 | 10 | 20 | 15 | 15 | 24 | 13 |  | R1a | 100.0 | [38] |
| Afg151 | Pashtun (Afghan) | R1a1a* -M198 | 16 | 11/14 | 13 | 30 | 25 | 11 | 11 | 13 | 14 | 11 | 10 | 20 | 15 | 17 | 24 | 13 |  | R1a | 93.8 | [38] |
| Afg113 | Pashtun (Afghan) | R1a1a* -M198 | 16 | 11/14 | 13 | 30 | 25 | 11 | 11 | 13 | 14 | 11 | 10 | 20 | 15 | 16 | 23 | 13 |  | R1a | 93.8 | [38] |
|  | Indian |  | 16 | 11/14 | 13 | 29 | 26 | 11 | 11 | 13 | 14 | 11 | 10 |  |  |  |  |  |  | NM | NDT | YHRD |
|  | Chinese |  | 16 | 11/14 | 13 | 29 | 26 | 11 | 11 | 13 | 14 | 11 | 10 |  |  |  |  |  |  | NM | NDT | YHRD |
|  | Chinese |  | 16 | 11/14 | 13 | 29 | 26 | 11 | 11 | 13 | 14 | 11 | 10 |  |  |  |  |  |  | NM | NDT | YHRD |
|  | Pakistani |  | 16 | 11/14 | 13 | 29 | 26 | 11 | 11 | 13 | 14 | 11 | 10 |  |  |  |  |  |  | NM | NDT | YHRD |
|  | Pakistani |  | 16 | 11/14 | 13 | 29 | 26 | 11 | 11 | 13 | 14 | 11 | 10 |  |  |  |  |  |  | NM | NDT | YHRD |
|  | Pakistani |  | 16 | 11/14 | 13 | 29 | 26 | 11 | 11 | 13 | 14 | 11 | 10 |  |  |  |  |  |  | NM | NDT | YHRD |
|  | Czech |  | 16 | 11/14 | 13 | 29 | 26 | 11 | 11 | 13 | 14 | 11 | 10 |  |  |  |  |  |  | NM | NDT | YHRD |
|  | Polish |  | 16 | 11/14 | 13 | 29 | 26 | 11 | 11 | 13 | 14 | 11 | 10 |  |  |  |  |  |  | NM | NDT | YHRD |
