# Supplementary material for: Molecular Genealogy of a Mongol Queen’s Family and Her Possible Kinship with Genghis Khan
Source: PLoS One. 2016 Sep 14;11(9):e0161622. doi: 10.1371/journal.pone.0161622 (PMC5023095; doi:10.1371/journal.pone.0161622)
Supplement: S8 Table — aThe probabilities expressed as percentages were rounded to two decimal places. bAllelotype of D21S11 in MN0126. UR: unrelated. (DOCX) [file pone.0161622.s018.docx]

**S8 Table. Kinship analysis among Tavan Tolgoi bodies using the Kinship Index Calculation program**

| **Kinship** | **Probability (%)**^a^ | | | | | | | | | | | | | | | | |
| --- | --- | --- | --- | --- | --- | --- | --- | --- | --- | --- | --- | --- | --- | --- | --- | --- | --- |
|  | **MN0104/ MN0105** | **MN0104/ MN0125** | **MN0104/ MN0126 (30/30**^b^**)** | **MN0104/ MN0126 (30/33.2**^b^**)** | **MN0104/ MN0124** | **MN0104/ MN0376** | **MN0105/ MN0125** | **MN0105/ MN0126** | **MN0105/ MN0124** | **MN0105/ MN0376** | **MN0125/ MN0126 (30/30**^b^ **)** | **MN0125/ MN0126 (30/33.2**^b^**)** | **MN0125/ MN0124** | **MN0125/ MN0376** | **MN0126/ MN0124** | **MN0126/ MN0376** | **MN0124/ MN0376** |
| **Parent-Child** | 0.0 | 100.0 | 0.0 | 0.0 | 0.0 | 0.0 | 0.0 | 0.0 | 0.0 | 0.0 | 0.0 | 100.0 | 0.0 | 0.0 | 0.0 | 0.0 | 0.0 |
| **Full Sibling** | 8.8 | 100.0 | 64.2 | 64.2 | 15.8 | 2.7 | 5.0 | 0.4 | 9.1 | 0.1 | 99.0 | 99.9 | 3.4 | 5.5 | 0.2 | 0.0 | 4.8 |
| **Half Sibling** | 29.8 | 100.0 | 77.3 | 77.3 | 53.5 | 39.4 | 20.9 | 8.4 | 40.9 | 6.7 | 97.0 | 99.5 | 26.7 | 55.6 | 7.1 | 2.1 | 45.8 |
| **First Cousin** | 45.1 | 99.6 | 72.2 | 72.2 | 57.2 | 52.0 | 38.7 | 28.0 | 50.1 | 27.9 | 90.3 | 96.5 | 43,7 | 63.4 | 26.3 | 17.8 | 52.8 |
| **UR** | ≤50.0 | ≤50.0 | ≤50.0 | ≤50.0 | ≤50.0 | ≤50.0 | ≤50.0 | ≤50.0 | ≤50.0 | ≤50.0 | ≤50.0 | ≤50.0 | ≤50.0 | ≤50.0 | ≤50.0 | ≤50.0 | ≤50.0 |
