# Supplementary material for: Molecular Genealogy of a Mongol Queen’s Family and Her Possible Kinship with Genghis Khan
Source: PLoS One. 2016 Sep 14;11(9):e0161622. doi: 10.1371/journal.pone.0161622 (PMC5023095; doi:10.1371/journal.pone.0161622)
Supplement: S9 Table — mtDNA haplogroups were determined by means of mtDNAmanager, a Web-based tool for the management and quality analysis of mtDNA sequences of control regions. Hp: haplogroup. (DOCX) [file pone.0161622.s019.docx]

**S9 Table. Distribution of mtDNA haplotypes and haplogroups of modern-day Mongolians**

| **Sample ID** | **Sex** | **HVR1 (15971-16410)** | **HVR2 (15-389)** | **Hp** |
| --- | --- | --- | --- | --- |
| MNNL0004 | M | 16223T 16311C 16362C | 73G 263G | D4/G |
| MNNL0005 | F | 16224C 16311C | 73G 146C 249d 263G | K2 |
| MNNL0006 | M | 16172C 16223T 16257A 16261T 16278T | 73G 150T 263G | N9a2 |
| MNNL0007 | F | 16092C 16223T 16325C 16362C 16399G | 73G 152C 204C 207A 235G | D1 |
| MNNL0008 | F | 16223T 16227G 16274A 16278T 16362C | 73G 152C 263G | D4g1 |
| MNNL0009 | M | 16223T 16227G 16278T 16362C | 73G 152C 263G | G2a1 |
| MNNL0010 | M | 16223T 16227G 16234T 16278T 16362C | 73G 152C 263G | G2a1 |
| MNNL0013 | F | 16185T 16223T 16260T 16298C | 73G 152C 195C 198T 249d 263G 275A | Z |
| MNNL0016 | M | 16223T 16298C 16327T | 73G 199C 263G | M8 |
| MNNL0020 | M | 16093C 16129A 16223T 16327T | 73G 152C 263G | R |
| MNNL0021 | M | 16129A 16223T 16362C | 73G 195C 263G | D4/G |
| MNNL0022 | M | 16223T 16227G 16278T 16362C | 73G 152C 207A 263G | G2a1 |
| MNNL0023 | M | 16093C 16223T 16290T 16362C | 73G 150T 263G | D4/G |
| MNNL0024 | M | 16223T 16298C 16327T | 73G 146C 151T 152C 207A 249d 263G | C4b |
| MNNL0026 | M | 16223T 16311C | 73G 152C 198T 263G 318C 326G | M11 |
| MNNL0027 | M | 16223T 16290T 16319A 16362C | 73G 152C 207A 235G | A4 |
| MNNL0031 | M | 16129A 16224C 16265G 16291T 16311C 16390A | 73G 150T 241G 263G | K |
| MNNL0034 | F | 16223T 16290T 16319A 16325C 16362C | 73G 152C 235G 263G | A4 |
| MNNL0035 | F | 16223T 16319A 16362C | 73G 239C 263G 297G | D4b |
| MNNL0036 | F | 16093C 16129A 16152C 16179T 16223T 16362C | 73G 263G | D4/G |
| MNNL0037 | M | 16126C 16146G 16153A 16240G 16294T 16296T | 73G 150T 204C 207A 263G | T2e |
| MNNL0038 | M | 16129A 16152C 16179T 16192T 16223T 16239T 16362C | 73G 263G | M7e |
| MNNL0039 | M | 16223T 16227G 16278T 16362C | 73G 195C 263G | G2a1 |
| MNNL0041 | M | 16224C 16311C 16362C | 73G 195C 263G | K |
| MNNL0042 | M | 16224C 16311C | 73G 146C 263G | K2 |
| MNNL0043 | M | *CRS* | 263G | H |
| MNNL0045 | M | 16180G 16223T 16288C 16291T 16298C 16327T | 73G 249d 263G | C5 |
| MNNL0048 | F | 16126C 16231C 16266T 16291T 16399G | 73G 146C 263G | Y1 |
| MNNL0049 | M | 16214T 16223T 16274A 16311C | 73G 146C 199C 263G | M7c |
| MNNL0051 | M | 16126C 16231C 16266T | 73G 146C 207A 263G | Y1 |
| MNNL0052 | M | 16223T 16234T 16278T 16311C 16362C | 73G 152C 263G | D4g1 |
| MNNL0053 | M | 16223T 16362C | 73G 204C 207A 263G | D4/G |
| MNNL0054 | M | 16093C 16129A 16223T 16298C 16327T | 73G 195C 249d 263G | C4a1 |
| MNNL0057 | F | 16174T 16223T 16278T 16298C 16327T 16368C | 73G 249d 263G | C |
| MNNL0058 | F | 16126C 16231C 16266T | 73G 146C 263G | Y1 |
| MNNL0059 | F | 16111T 16129A 16223T 16257A 16261T | 73G 150T 263G | N9a1 |
| MNNL0061 | F | 16171G 16223T 16278T 16298C 16327T 16344T 16357C | 73G 249d 263G | C4a |
| MNNL0062 | M | 16093C 16223T 16298C 16327T | 73G 150T 249d 263G | C |
| MNNL0063 | M | 16224C 16245T 16292T 16362C | 73G 263G | D4c |
| MNNL0064 | M | 16223T 16362C | 73G 195C 263G 298T | D4 |
| MNNL0066 | F | 16223T 16227G 16278T 16362C | 73G 152C 207A 263G | G2a1 |
| MNNL0067 | F | 16129A 16185T 16223T 16224C 16260T 16298C | 73G 151T 152C 249d 263G | Z |
| MNNL0068 | F | 16176T 16223T 16290T 16319A 16362C | 73G 152C 235G 263G | A4 |
| MNNL0071 | M | 16223T 16274A 16362C | 73G 152C 263G | D4/G |
| MNNL0072 | M | 16185T 16223T 16260T 16293G 16298C | 73G 249d 263G | CZ |
| MNNL0074 | F | 16129A 16150T 16298C 16327T | 73G 195C 249d 263G | C |
| MNNL0078 | F | 16145A 16192T 16243C 16304C 16309G 16390A | 73G 183G 263G | R9b |
| MNNL0080 | F | 16223T 16362C | 73G 195C 198T 263G | D4p |
| MNNL0096 | M | 16093C 16193T 16223T 16311C 16357C | 73G 146C 189G 199C 263G | M7c |
| MNNL0102 | F | 16093C 16203G 16291T 16304C | 73G 249d 263G | F2a |
| MNNL0108 | F | 16093C 16223T 16232T 16290T 16362C | 73G 195C 263G | D4o |
| MNNL0109 | M | 16129A 16223T 16362C | 73G 152C 263G | D4a |
| MNNL0113 | M | 16093C 16223T 16232T 16290T 16362C | 73G 195C 263G 297G | D4o |
| MNNL0115 | F | 16223T 16227G 16278T 16362C | 73G 152C 207A 263G | G2a1 |
