# Supplementary material for: Molecular Genealogy of a Mongol Queen’s Family and Her Possible Kinship with Genghis Khan
Source: PLoS One. 2016 Sep 14;11(9):e0161622. doi: 10.1371/journal.pone.0161622 (PMC5023095; doi:10.1371/journal.pone.0161622)
Supplement: S10 Table — aEthnic distribution of individuals subjected to buccal swab analysis. bPercentage of ethnic groups in all Mongolians was presented based on Mongolia population census data (2010). (DOCX) [file pone.0161622.s020.docx]

**S10 Table. Ethnic distribution of modern-day Mongolians selected to determine the haplogroups of mtDNA and Y chromosome**

| **Ethnic group** | **Sample (%)^a^** | **Mongolian^b^** |
| --- | --- | --- |
| Khalkh | 107 (87.7) | 82.4 |
| Khazakh | 4 (3.3) | 3.86 |
| Uriankhai | 4 (3.3) | 1.01 |
| Oold | 3 (2.5) | 0.59 |
| Dorvod | 2 (1.6) | 2.75 |
| Buriad | 1 (0.8) | 1.71 |
| Zakhchin | 1 (0.8) | 1.25 |
| Others | 0 | 6.43 |
